# Automatic grading for Arabic short answer questions using optimized deep learning model

**DOI:** 10.1371/journal.pone.0272269

**Published:** 2022-08-02

**Authors:** Mustafa Abdul Salam, Mohamed Abd El-Fatah, Naglaa Fathy Hassan

**Affiliations:** 1 Artificial intelligence Dept., Faculty of Computers and Artificial Intelligence, Benha University, Banha, Egypt; 2 Faculty of Computer Studies, Arab open University, Nasr City, Egypt; 3 Information System Dept., Computers and Information Faculty - Benha University, Banha, Egypt; 4 Information and Operations Dept., National Center for Examinations and Educational Evaluation (NCEEE), Cairo, Egypt; University of Eastern Finland: Ita-Suomen yliopisto, FINLAND

## Abstract

Auto-grading of short answer questions is considered a challenging problem in the processing of natural language. It requires a system to comprehend the free text answers to automatically assign a grade for a student answer compared to one or more model answers. This paper suggests an optimized deep learning model for grading short-answer questions automatically by using various sizes of datasets collected in the Science subject for students in seventh grade in Egypt. The proposed system is a hybrid approach that optimizes a deep learning technique called LSTM (Long Short Term Memory) with a recent optimization algorithm called a Grey Wolf Optimizer (GWO). The GWO is employed to optimize the LSTM by selecting the best dropout and recurrent dropout rates of LSTM hyperparameters rather than manual choice. Using GWO makes the LSTM model more generalized and can also avoid the problem of overfitting in forecasting the students’ scores to improve the learning process and save instructors’ time and effort. The model’s performance is measured in terms of the Root Mean Squared Error (RMSE), the Pearson correlation coefficient, and R-Square. According to the simulation results, the hybrid GWO with the LSTM model ensured the best performance and outperformed the classical LSTM model and other compared models such that it had the highest Pearson correlation coefficient value, the lowest RMSE value, and the best R square value in all experiments, but higher training time than the traditional deep learning model.

## 1 Introduction

The learner’s knowledge evaluation is observed as a form of the major aspects of the teaching process, such as it is an estimation of the student’s performance in examinations and other activities. In the traditional evaluation, a human grader provides feedback for the students on their answers to questions. The bias and views on the free text questions in human grading may influence the overall grade of the student. Furthermore, with a large increase in the number of students and rapid technological development, automatic/computerized assessment was required to achieve fairness in the grading process, faster checking of questions, and to reduce time cost and a lot of human effort in the assessment process. Some forms of assessment such as Choice from Various, Correct or Incorrect, Corresponding and Fill in the blank questions may be easily scored by a system such as its design and implementation is a simple task, but there are answers made of free text which raises the student‘s writing skills and decreases the cheating process rate. Grading essay responses and short answers require textual analysis and understanding, so the implementation and the design of it remain a difficult and complicated task. Automatic Essay Scoring is focused on grading essay questions such that the answers questions are long without model answers delivered and related to analyzing the spelling, grammar, and coherency of sentences. On the other hand, the grading of the short answers is focused on grading short answers that contain two to three sentences of length with the existence of one or more model answers. The main concern is to grade a learner’s answer with regard to the model answer. Sentence structure and coherency are not of concern in many approaches dealing with the grading of short answers. Various researchers consider grading the short answer in place of a challenging issue in NLP (Natural Language Processing) such that it is a group of techniques used to derive meaning from text data. So, NLP is based on textual similarity evaluation. [1] showed and discussed the popular methods used in evaluating short text similarity, such that they examined and compared each other and demonstrated how the approaches used in short text similarity evaluation changed over time. While some researchers, such as the work of [2], used several words overlapping methods to count the similar words between the learner’s answer and the model answer, this study concluded that the algorithm of the overlapping word cannot overcome the semantic similarity problems, such as some students can express the correct answer in words different than the model answer keywords. As a result, this study navigates text-similarity measurements and discusses many works related to short answer question auto-grading while keeping in mind that the correct answer may not contain the same words as the model answer.

### 1.1 Contributions

The primary contributions of this paper are:

- Investigating text similarity measurements.
- Utilizing algorithms of machine learning and techniques of deep learning to predict the student grade in Arabic Short Answer questions automatically, as there is a shortage of research on the Arabic language short answer auto-grading, which requires handling the language of Arabic in NLP and it is a difficult task because of its complexity and because it is an actual rich language.
- Blending the Grey Wolf Optimization technique with LSTM for optimizing the system performance, as the hybrid LSTM-GWO model results showed the best accuracy compared with other models.

### 1.2 Paper organization

The remainder of this paper is structured as follows. Section two describes works that are related to the short answer questions auto-grading. Section three presents the text preprocessing techniques. Moreover, the similarity measurements are introduced in section four. Section five describes the proposed work accompanied by an explanation of the GWO technique. While the experimental results and discussion are presented in section six. Finally, section seven concludes our work and indicates the future work.

## 2 Related work

In the literature review, many of the techniques were used in automatic scoring. In recent years, such techniques within machine learning and deep learning approaches have been used to present strong models in short answer questions auto-grading such as:

**Wael H Gomaa et al**. [3] have used a BoW (Bag of Words) as an unsupervised approach that does not need predefined patterns or scoring rules. They separately tested the string-and corpus-based algorithms to measure the similarity between model response and student response and combined them to reach the best value of correlation is 0.504 from combining the similarity values of N-gram and Disco1. The dataset was made up of 81 questions and 2273 answers for students.

**Al-awaida Odeh et al**. [4] proposed a system for automatically grading an Arabic essay using the SVM (support vector machine) mechanism to select features from student responses and model responses after preprocessing. The research used cosine similarity to define the grade of a student response via finding out the similarity degree among the student response and previously determined response models. The application on Arabic WordNet was proposed to increase the Automated Essay System’s accuracy to match the human score. The used dataset contains 40 questions with a 120 answer model according to helmet ASAP from Kaggle datasets. The system was implemented in python.

**K Surya et al**. [5] had investigated some common deep learning models for the short answer scoring task. They used: (1) Character level CNN. (2) Word-level CNN. (3) Word-level bi-LSTM. (4) BERT. The comparison between these models resulted in perfect BERT results over the other models’ outcomes, and the LSTM model outcomes are perfect than CNN models’ results. They tested the reliability of the models by modifying a few examples from the data that were scored correctly by all the models. They found that the RNN model performed well over the other models when introducing spelling mistakes and replacing a few words with a synonym, but BERT performed better when the responses were paraphrased.

**Neethu George et al**. [6] presented a deep learning and NLP (natural language processing) based descriptive response checking and grading system. This system, which consists of an embedding layer, LSTM (Long Short Term Memory) layer, dropout layer, and dense layer, was called the “deep descriptive answer scoring model”. The text-relevant features in the response scripts are extracted and converted into glove vector indexes during the preprocessing stage. Then, the glove index corresponding to one word is converted into glove vectors by the embedding layer. LSTM-the recurrent neural network layer sequentially receives each word’s glove vectors in the response and converts this into a semantic representation. So, the semantic representation of the entire response is the embedding vector that corresponds to the last word in the response. In the dense layer, the softmax activation function will predict the one-hot encoded score for each response.

**Lucas Busatta Galhardi et al**. [7] followed guidelines to conduct a systematic review for the automatic grading of short responses such that the emphasis was on the research which applied machine learning techniques such as decision tree, support vector machine, k-nearest neighbors, and linear regression. They investigated the importance of the automatic grading of short responses in the education domain. The suggested review was based on the answers to 4 research questions executed on forty-four papers.

**Brian Riordan et al**. [8] used LSTM and CNN deep neural approaches for the short answer scoring. Each response word token is converted to embeddings. Then, features are extracted from the embeddings by the layer of CNN (Convolutional Neural Network). This output represents the input to LSTM (a Long Short Term Memory) layer. The LSTM hidden states are aggregated in either a “mean-over-time” (MoT) layer or an attention layer. The aggregation layer output is a single vector that represents an input to a fully connected layer that computes regression or classification. The experiments offered the strength of the neural networks over the non-neural baseline system in scoring short answer content.

**Stephen Pulman et al**. [9] examined the techniques of CL (computational linguistics) in automated short responses grading such that they investigated machine learning approaches such as DTL (decision tree learning), ILP (inductive logic programming), and Naive Bayesian learning and showed how these methods are performed well than when using manual patterns in extract information. Also, they discussed the issues that impact the accuracy estimation for automated systems, such as inconsistencies in responses. They conducted two experiments, one on non-annotated data and the other on annotated data.

**Udit Kr Chakraborty et al**. [10] proposed a system to automatic the assessment of short responses based on clusters of a rough concept, in which they massed keywords from the model responses group to form words’ clusters. They used the clusters of rough sets together with functions of fuzzy membership in the responses’ evaluation. The system was tested with a dataset in English which consists of four questions and 281 single-sentence answers. The results showed correlation values were better than those of human evaluators, and the performance was enhanced compared to assessment systems based on LAS (Latent Semantic Analysis) and link grammar.

**Pranjal Patil et al**. [11] developed a hybrid model for auto-grading short answers. The proposed model consists of two sub-models: one for sentence modeling and the other for measuring semantic similarity. They used a Siamese neural network composed of 4 sub-networks to represent sentences such that each sub-network has 3 layers, in which vectors of word embedding are provided to a Bi-LSTMs which are initialized with normal weights such that the output of a Bi-LSTMs is a vector delivered into an attention layer to offer importance to diverse words of the sentences. They proposed a fully connected network with a logistic regression layer to model similarity to calculate the student’s answer correctness. They used a dataset with 135 questions in different fields of physical sciences such that each question has a reference short response and 36 students’ answers.

**Michael Mohler et al**. [12] examined unsupervised techniques and made comparative assessments of text-similarity measurements based on knowledge and corpus, for automatic short response grading. They assessed the impact of the domain and size on the corpus-based measurement performance. Also, they introduced an automated feedback method with students’ responses to improve the system performance through handling the problem of correct student responses which are not similar to the model answer. The used dataset contained 630 student responses for 21 questions. The results offered that the measurements based on knowledge (WordNet shortest path and Jiang … Conrath) and the measurements based on the corpus (such as LSA and ESA) have the best performance, such that LSA achieved the best correlation value of 0.5099, which was calculated by comparing the degree assigned to every student’s answer with the actual grade, and 0.6735, which was computed by comparing total student grades with the actual totals of degrees per student.

**Sarah Hassan et al**. [13] introduced paragraph embedding models for short answer automatic grading. They used two methods to generate paragraph embedding, one to compute the sum of pre-trained word vectors for words in the response, and another to train a deep learning model to directly deduce the paragraph vector of a certain answer. Word2Vec, GloVe, Fasttext, and Elmo were the word embedding techniques, while Doc2vec, InferSent, and Skip-Thoughts were used for paragraph embedding. They conducted the experiments using a dataset of 2273 short responses. They examined different models for automatic short response grading such that they generated a paragraph vector for each answer, then used cosine similarity to determine the similarity between the paragraph vectors for each couple of student responses and model answers. After that, they trained a regression-classifier for expecting students’ grades and calculated accuracy where the training doc2vec model achieved the best correlation value of 0.569 and the best RMSE value: 0.797.

**Md Arafat Sultan et al**. [14] presented a high-accuracy, simple design, and fast run-time system for short answer grading. They computed three features of text similarity: Alignment, Semantic Vector Similarity, and cosine similarity. This study augments the text-similarity features with question demoting (such as eliminating words that appear in the text of the question from both the student answer and the reference response) and term weighting techniques (such that determine important answer words) to generate new features. The final feature was the proportion of the number of words in the student answer to in the reference response. They trained two supervised models with the final feature. The results showed improvements in performance above the state of art.

**Ayad R Abbas et al**. [15] suggested an automated grading system for web-based learning context Arabic essays. The vectors space model (VSM) and LSI (latent semantic indexing) were used to measure the similarity degree between the student’s essay and the essay the instructor wrote before. The experiments were executed on one question with four model answers and 30 student responses. The results showed that the proposed system evaluation is more similar to the judgments of instructors which led to performance improvement.

**Laurie Cutrone et al**. [16] examined the recent research in NLP to implement a computerized assessment system for automatic grading short responses. The developed system was a component-based architecture to support Learning Management Systems such that the focus was on the semantic meaning of the responses of the student. The system was utilized to process responses with a single sentence, eliminating grammar and spelling errors. The used tool was WordNet.NET.

**Swarnadeep Saha et al**. [17] mixed the features of token and sentence level to automate short response marking. The system was proposed to overcome the accuracy limitation in the features of token level and also the sentence level features domain dependence. They calculated the features of sentence-level after getting the sentence embeddings of the student response, model answer, and question. The features of token level are extracted through word overlap, HoPS which is a partial similarity histogram, HoPSTags (HoPS with part-of-speech (POS) Tags), and question types. The representation of the final feature is a total summation of the features of sentence and token level. The experimental results from using three datasets were better than on the state-of-art.

**Xi Yang et al**. [18] proposed a system for auto-grading short responses of students using modern machine learning techniques. They built 3 models as classifiers of the SVM (Support Vector Machine), the Long Short Term Memory (LSTM) algorithm, and blended the SVM and LSTM into one module. The domain of study was comprehension of Chinese reading. The study was conducted on one question with 534 student answers. The overall kappa was 0.755 and the accuracy of the combination between SVM and LSTM in one model was better than using SVM and LSTM separately.

**Michael Mohler et al**. [19] enhanced BOW (bag-of-words) approaches to short response grading based on machine learning methods. They blended various graph alignment features in a three-stage approach to short response grading such as they produced an overall grade based on the alignment grades and semantic BOW similarity measures. They used SVM (Support Vector machines) to create a combined real number grade. Finally, they built an Isotonic Regression (IR) model to convert the output grades onto the original [0, 5] scale used by the annotators.

**Ahmed Ezzat Magooda et al**. [20] proposed a vector-based short response grading system. They used: 1) similarity measures: Block distance, Jiang Conrath, Lesk, DISCO, and 2)Vector Representations: Word2Vec, GloVe, and Sense Aware Vectors to calculate word-to-word similarity. The sentence similarity is calculated through three models: Text-to-text model, Vector summation model, Min-Max Additive model. The system achieved better results and outperformed the state of art systems on four datasets.

**Neslihan Süzen et al**. [21] proposed an automatic marking system to predict student marks in short-response questions based on data mining techniques used to calculate the similarity among the model response and student responses. For text representation, they used the BoW (Bag of Words) model. The k-means algorithm was applied for grouping student responses into clusters such that each cluster has the same score and feedback on each response in a cluster. The experiments were conducted on an introductory computer science dataset at North Texas University.

**Steven Burrows et al**. [22] presented an exhaustive survey of automatically short response grading systems and research dependent on components and historical analysis. The analysis of components examines 6 familiar dimensions which include: natural language processing, data sets, model construction, scoring models, model assessment, and efficiency. Through the historical analysis, 35 automatic short response grading systems were identified in 5 eras which include: concept mapping, extraction of information, methods based on a corpus, machine learning, and assessment. The study resulted in that time of assessment is the latest trend in automatic short response grading.

**Fatma Elghannam et al**. [23] proposed a semantic similarity measurement automatically between short texts of the Arabic language. The researcher combined the lexical similarity measurements with the semantic distribution measures to achieve the best accuracy value of 97%, which is better than depending only on calculations of word distribution similarity whose accuracy reached 93%.

**Wael Hassan Gomaa et al**. [24] suggested an Arabic free-text response automatic assessment by using multiple measures of text similarity separately and combining them. They created a data set of 610 short answers from students. The gained values of similarity were scaled by applying K-means clustering. They conducted experiments to handle String-Based Similarity 4 types, including DL, LCS, Character-based N-gram, and Word-based N-gram, and Corpus-Based Similarity 2 methods, DISCO1 and DISCO2. After that, they translated from Arabic to English and used the English WordNet to test the Knowledge-Based measures. Finally, they used WEKA to combine various similarity measures to improve the correlation results to 0.83 and decrease RMSE to 0.75. The results were very adjacent to manual scoring.

**Goutam Majumder et al**. [25] developed a method for determining the similarity of semantic texts through using WordNet taxonomy and merging information content with a Trigram-based language model. Such as, when the method is unable to find the information content (IC) value, they use the trigram frequency count in the corpus as the IC value. The SemEval training 2015 dataset was used to evaluate the proposed system, and the experimental results achieved a 0.885 similarity score.

**Tuanji Gong et al**. [26] proposed an attention-based deep model which integrates pre-trained word embedding techniques such as LSTM (Long Short Term Memory) with a bidirectional RNN unit and an attention mechanism for automatic short answer scoring. The authors conducted two experiments to compare the suggested model with baseline models such as traditional linear regression and latent semantic analysis, and the results showed the suggested model achieved a 10% increase in performance.

**Abdulaziz Shehab et al**. [27] had presented a comparison between various algorithms of text similarity to build a system for automatic grading of Arabic essays. They used corpus and string algorithms separately. The string-based similarity algorithms were N-gram and Damerau-Levenshtein (DL). The algorithms of corpus-based similarity were DISCO and LSA (Latent Semantic Analysis). They used a data set of 210 student responses, and the study found that the n-gram outperformed other algorithms in Arabic essay auto-grading.

**Fawaz S Al-Anzi et al**. [28] examined the cosine similarity measurement achievement for the classification of Arabic text such that they use Latent Semantic Indexing to generate the features of the dataset, which consists of 4000 documents. Then, they made an empirical comparison among cosine similarity assessment and various classifiers from machine learning such as k-Nearest Neighbor, classification trees, SVM, neural networks, Naïve Bayes, LR, Random Forest, and CN2 induction Rules. The research results revealed that similarity measurement by cosine is an ideal method for the classification of Arabic text and that k-NN (cosine-based measurement) and SVM perform best among the other classifiers. The proposed system was implemented using Python.

### 2.1 Discussion and related works

Based on previous studies, the use of optimization techniques in the automatic grading of short answer questions has been ignored. This proposed system uses GWO to achieve a fair and accurate assessment of students’ answers to Arabic short answer questions, because GWO is one of the most recent optimization algorithms, has rapidly progressed as a swarm intelligence-based technique, and is characterized by flexibility, making it the best choice for other optimization algorithms such as GA (Genetic Algorithm) and ACO (Ant Colony Optimization) to select the optimal features and use them in LSTM to optimize performance, which enhances the learning process.

Table 1 depicts briefly some of the related and significant work from the literature review in automatic grading.

**Table 1:** A summary of related work.

Author	Technique	Advantages	Limitations
Wael H Gomaa et al. (2012)	• Bag of Words -Character-based and Term-based distance measures - DISCO1 and DISCO2	• Easy BOW implementation - Best Correlation value is 0.504 from mixing N-gram and Disco1.	• Available only for English - Not support the Arabic language
Al-awaida Odeh et al. (2019)	• support vector machine - cosine similarity - Arabic WordNet	• Easily implemented in python - Improved accuracy to match human score	• Small dataset - Support only the Arabic language
K Surya et al. (2019)	Character level CNN - Word level CNN - Word level bi-LSTM and BERT	• Used modern deep learning models such as BERT - Simple and efficient architecture	• More computational resources are needed for training
Neethu George et al. (2019)	• LSTM - Machine Learning - RNN	• Efficient for grading any descriptive answer	• Lack of details about the dataset
Brian Riordan et al. (2017)	• LSTM and CNN	outperformed the non-neural system	Performance varies when the same optimal parameters are used
Udit Kr Chakraborty et al. (2016)	• Fuzzy membership functions with rough set theory	High accuracy compared with LAS (Latent Semantic Analysis)	only applied to single sentence responses - small dataset
Pranjal Patil et al. (2018)	• Siamese neural network	Large dataset	Misclassification of some students’ responses - low detection of similarity
Sarah Hassan et al. (2018)	• Word2Vec - GloVe - Fasttext - Elmo - Doc2vec - InferSent - Skip-Thoughts - and Cosine similarity	Answer can be extended to be one paragraph answer	small dataset
Md Arafat Sultan et al. (2016)	• Alignment - Semantic Vector Similarity - and cosine similarity	Several benchmarks - simplicity - speed - high accuracy	Free term weighting methods
Swarnadeep Saha et al. (2018)	• Histogram of Partial Similarities or HoPS and its extension to part-of-speech tags (HoPSTags)	• Generalizability - effectiveness - several benchmarks	Non-overlapping
Neslihan Süzen et al. (2018)	• Bag of Words - k-means algorithm	Fast feedback - enhance consistency in grading	Not reliable
Goutam Majumder et al. (2017)	• WordNet Taxonomy - Trigram - Information Content	High value for correlation coefficient	few related works and less detail in the results discussion
Tuanji Gong et al. (2019)	• Word embedding - attention mechanism - and Bidirectional RNN with LSTM unit	Two or more reference responses - an attention mechanism that improves the outcomes	Fewer evaluations
Proposed work (2022)	• N-Gram - Arabic WordNet - LSA - Word2vec - LSTM - MaLSTM - SVM - and GWO	large dataset - accuracy - Effectiveness - fast - reliable - optimized performance	Free recent federated deep learning models - limited to only the Arabic language

## 3 Text preprocessing

Text preprocessing is the main step in Natural Language Processing (NLP) tasks. It converts the text into a more predictable and analyzable form so that the algorithms of machine learning will perform superior. Some preprocessing steps should be followed before measuring the text-similarity to get more accurate results. Text preprocessing techniques are: - tokenization, stop-words removal, normalization, stemming or lemmatization, and part-of-speech.

### 3.1 Tokenization and stop-words removal

Tokenization can be defined as the procedure of dividing or breaking a text into a token list. Tokens could be a word is a token in an expression or a phrase is a token in a paragraph.

Meaningless words are denoted as stop words in NLP. Stop words are a set of repeatedly used words in a language that don’t contribute to the document semantics and have no extra value (e.g. pronouns and prepositions). In English, the words “a”, “the”, “us”, or “our” are examples of stop words, while in Arabic, some stop words are “أو”, “إذا”, “كل”, “بين”, “كما”, “ما”, “هذه” etc. By eliminating words of low information from the text, the concentration will be on the keywords instead and also reduces the corpus size, which leads to higher efficiency. [29] presented a statistical approach to extract a list of Arabic stop-words.

### 3.2 Normalization

**Fig 1:**
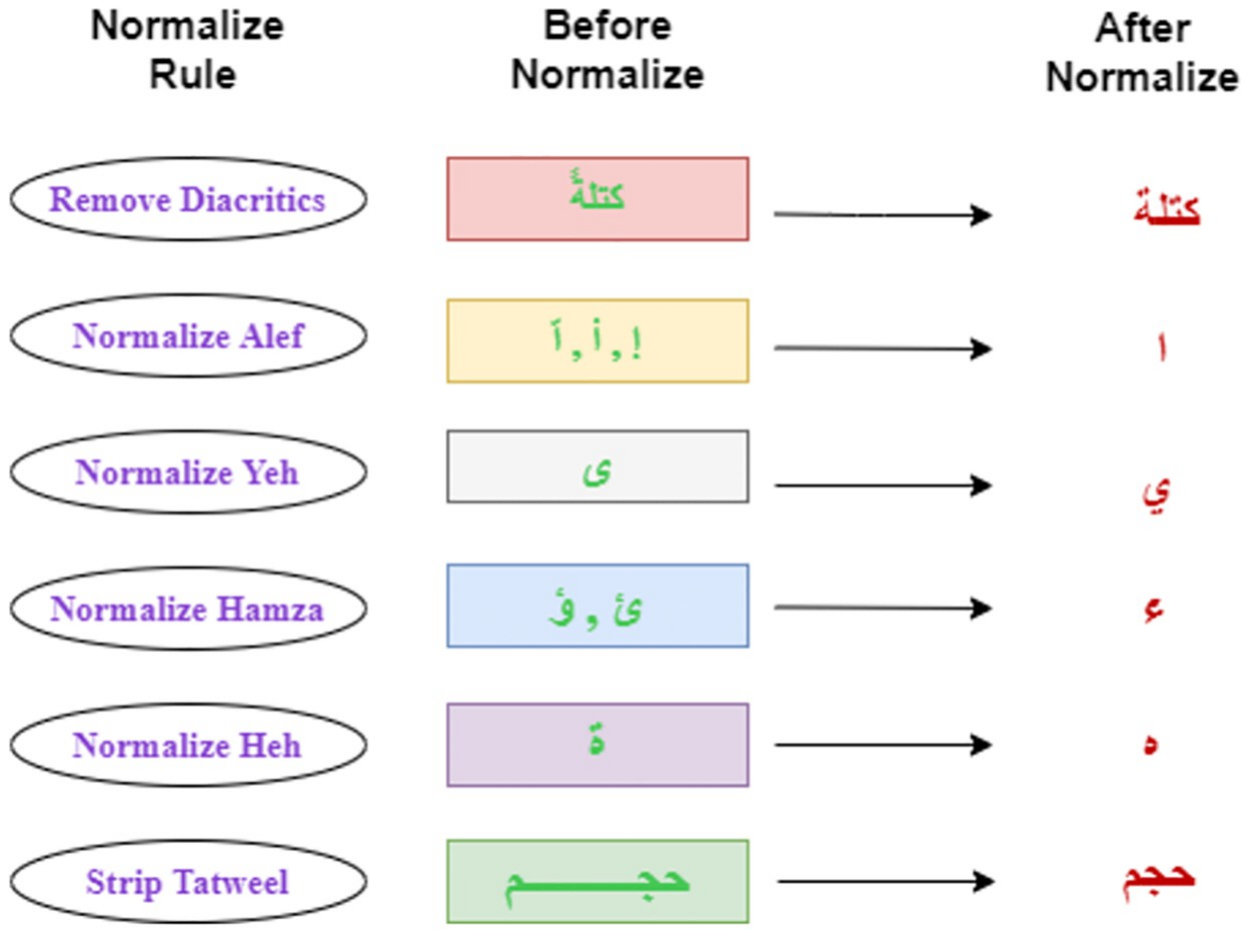
Rules of normalization.

The procedure of normalizing a text is the transformation of a text into an ordinary (standard) form. The words “2moro” and “tomrw” for example, can be converted into their standard form, “tomorrow”. The mapping of near-exact words is another case, such as “key-words”, “key words”, and “keywords” to just “keywords”. [30] used normalization with the method of frequency ratio accumulation to enhance Arabic text classification accuracy. The normalization rules are depicted with examples in Fig 1.

### 3.3 Stemming or lemmatization

Stemming has a significant role through NLP when it comes to eliminating suffixes or prefixes. Stemming sometimes leads to improper interpretation and errors in the spelling, although lemmatization takes the context into account and transforms it into its basic meaningful form such that it returns a word into its basic or root form called a lemma. There are two mechanisms of stemming: Tashaphyne stemmer and ISRI stemmer, which assist in improving the Arabic text classification accuracy [30]. For example, terms (افتضربوننى, تستلزم, مكتبة, محامون) will convert into (آفتضربون, لزم, كتب, حام) in the ISRI stemmer but will convert into (ضرب, لزم, كتب, حام) in the Tashaphyne stemmer.

### 3.4 Parts-of-speech (POS) tagging

The procedure of assigning one of the speech parts to a certain word is called Parts-Of-Speech (POS) tagging. Generally, POS tagging is a method of marking each word in a phrase with its proper section of speech. This is often a simpler way for school children to identify words like verbs, adverbs, nouns, pronouns, adjectives, conjunctions, etc. [31] generated a set of syntax-based rules at the phrase level which introduces important enhancements in the accuracy of tagging for speech parts that lead to an increase in the accuracy and quality and accuracy of NLP methods.

## 4 Similarity measurement

Text similarity should define how two “adjacent” parts of the text are in both surface proximity (lexical similarity) and significance (semantic similarity). Methods of text similarity play a role in research fields, like machine translation, information retrieval, summarization, etc. Many approaches for measuring word similarity rely on the distributional hypothesis (DH) [32]. Text similarity measurements are string-based similarity, semantic-based similarity, and hybrid-based similarity.

### 4.1 String-based similarity

It matches two streams of characters and determines the similarity mark based on the string corresponding to the two strings regardless of their meaning. The techniques for measuring string similarity are N-gram, which expects the word “happening” to be built on the occurrence of its (N-1) previous words or characters, a Jaccard similarity that calculates the similarity between sets by dividing the shared elements by the total elements of the two sets, a Dice’s coefficient, and the longest common sub-string, which is a contiguous series of letters that is mutual between two arrangements by removing particular features without modifying the order of the residual features, Hamming distance and a Generalized Hamming Distance [33], Levenshtein distance [34], Needleman-Wunsch distance that divides a problem into a chain of smaller problems and uses the smaller problem solutions to find an optimal solution to the problem, Smith-Waterman distance, which defines similar areas among sequences by comparing pieces of all probable lengths, and Jaro Winkler distance that only finds duplication and not much else.

### 4.2 Semantic-based similarity

**Fig 2:**
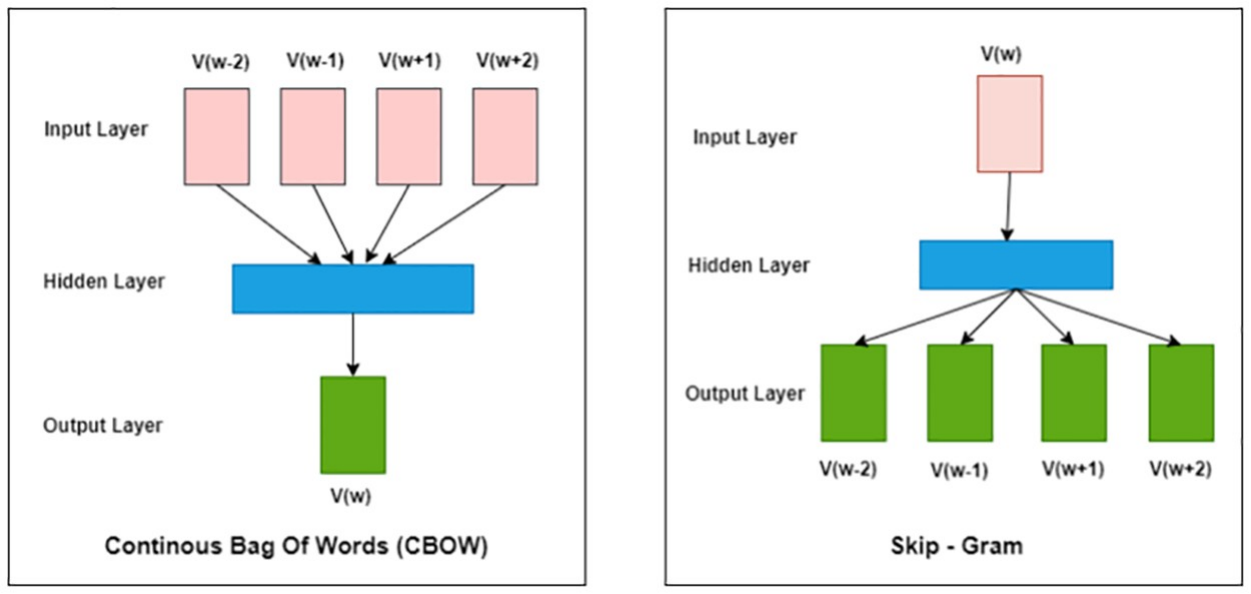
The Word2vec models: CBOW and Skip-gram.

Semantic-based similarity is a metric defined by a set of terms or documents. The comparison between them depends on the semantic content or agreement of their meaning. The algorithms used in semantic-based similarity are as follows:

1. Corpus-based similarity: it relies on information entirely acquired through processing large corpora, which is the plural of corpus (a huge collection of text documents), to build a knowledge space, which is used afterward to compute the relations between words and documents. [35] introduced a Vector Space Model updated version to measure the shortened text similarity by focusing on corpus-based methods.The common models used in corpus-based are as follows:
  - Latent semantic analysis (LSA): is a representation statistical model that utilizes vector averages in semantic space to estimate the similarity between texts or terms [36]. LSA constructs a term-document matrix using the raw data to list terms in rows and documents in columns in which every cell indicates how often a term happens in this document, then performs an SVD (Singular Value Decomposition) to decrease the number of rows, and finally, documents are compared by calculating the cosine angle between the two vectors made by any two columns. [37] used LSA to evaluate human similarity ratings of word pairs. [38] had used LSA to reveal hidden relationships among the documents and the terms.
  - Pointwise mutual information (PMI): was proposed as an unsupervised measurement to evaluate the semantic similarity by [39]. It depends on word co-occurrence via usage counts gathered from very large corpora such as the web.
  - Extracting distributionally similar words using co-occurrence (DISCO): calculates the distributional similarity among words such that seeing words that have similar meanings happens in a similar circumstance. The two key similarity measurements in DISCO are DISCO1 to calculate the similarity of first-order distributionally among two input words dependent on their clustering sets, and DISCO2 to calculate the similarity of second-order among two input words dependent on their sets of similar words.
2. Knowledge-based similarity: measurements are built on finding the amount of similarity among words using information acquired from lexical resources or semantic networks such as WordNet. The calculated semantic similarity reflects how the two words are related by the semantic properties of the two words, not by the structure of words. The popular knowledge-based similarity measurements are Res, JiangConrath, Lin, Lch, Shortest Path, Wup, Lesk, and Hso [40].
3. Vector-based Similarity: models represent the meaning of words in NLP and measure their semantic similarity through calculating their distance in the vector space, which is a statistical model that represents documents by way of vectors of identifiers such as index terms. Cosine similarity is generally used for measuring distance through the angle cosine among two vectors defines if the two vectors are referring to approximately the same trend. A score close to 1 indicates similarity, while a score close to 0 means they are completely unrelated. The similarity of cosine for two vectors, D and K, is calculated with Eq 1.
  $$\begin{array}{c} \text{cos}\left(\theta \right)=\frac{D.K}{∥D∥∥K∥}=\frac{\sum_{i=1}^{n}D_{i}K_{i}}{\sqrt{\sum D_{i}^{2}}\sqrt{\sum K_{i}^{2}}} \end{array}$$ (1)
  [41] Proposed two methods to maximize a particular weighted cosine similarity measure. Even though cosine similarity has some problems [42], it is still the most popular measure. The APSyn (Average Precision for Synonymy) measure was suggested by [43] and assessed by [44] to handle the limitations in the vector cosine. Terms of near meaning are also close to the space of the vector and vice-versa. The model of vector space can be generated via:
  - Term frequency-inverse document frequency (TF-IDF): is a procedure that weighs a word to mirror the word’s importance in regards to a corpus using the times’ number that a word happens in a document. [45] suggested SEMTFIDF as a metric of the similarity of semantic utilizing TF/IDF in estimating the similarity of semantic among texts. [46] proposed an embedded-based feature selection algorithm to classify the web pages using the web page side information, headings, and link texts and not fully dependent on term frequencies.
  - Word Embedding: represents a data of text, whether words or sentences, in real number vectors where the words that have the same meaning have a similar representation [47]. [48] suggested a measure based on rank for word embedding. Some of the models regarding word embedding are a Word2vec technique, which is utilized in NLP through training a two-layer neural network model on a huge text corpus to learn associations between each distinct corpus word and create a space of vectors such that the Word2Vec works in either the continuous bag of words (CBOW) or the Skip-gram [49]. Fig 2 shows that CBOW utilizes the context to forecast the aimed word and the Skip-gram utilizes a word to forecast the aimed context.
    While GloVe is used to get a global vector representation for words in a corpus such as [50, 51], as well as AraVec is an open-source project for the representation of distributed words that was pre-trained and designed to provide free usage and robust word embedding methods for the community research of Arabic NLP such that [52] described the steps to develop such models.

### 4.3 Hybrid-based similarity

Hybrid methods are based on combining two or more methods to benefit from their advantages and fill the gaps in each. [53] combined 16 standard measurements to show that mixture measurements outperformed single measurements, while [54] developed a system called yiGou using the model of support vector machine with literal resemblance, shallow syntactic resemblance, similarity using WordNet, and LSA to predict the total semantic similarity for two English phrases, as well as [55] developed a method for determining the semantic resemblance of short texts built on six knowledge-based and two corpus-based similarity measurements, [56] introduced the LIPN-CORE system such that the model of support vector regression was used to combine different semantic, syntactic, and lexical measures of text similarity, [57] suggested a method of STS (Semantic Text Similarity) through merging the LCS measurement and a semantic word resemblance based on a corpus, and [58] proposed a method for enhancing STS with PEA (Phrase Entity Alignment) using a corpus-based and knowledge-based similarity.

### 4.4 Machine learning and deep learning techniques

Machine learning is applied in many areas, such as [59] proposed a recommendation system for identifying the best location to start a business, [60] detected the fake news through using an ensemble model that is composed of three models of machine learning. There are machine learning algorithms and deep learning techniques that are employed to measure semantic similarity in short texts, such as:

1. Support vector machine (SVM): is employed for many purposes, such as regression, anomaly detection, or classification. The SVM basis is to discover the finest hyperplane which splits a data set into two groups such that the data points that are nearby to the hyperplane are called support vectors, while the space between the hyperplane and support vectors is named a margin. The SVM seeks to discover the hyperplane with the greatest margin to accomplish a good separation, since in general, the greater the margin, the lesser the classifier’s generalization error. [61] optimized the Mahalanobis similarity metric directly for minimizing the SVM classification error. [62] utilized the SVM approach to present an efficient system that measures the semantic textual similarity to both sentence pairs of English and Spanish.
2. Artificial neural network (ANN): A neural network (NN) is an information processing system that seeks to mimic human brain functionality by creating a large network of interconnected elements inspired by human neurons. A NN comprises three layers of input, hidden, and output layer. A NN is called a DNN (deep neural network) when it has layers of hidden exceeds one such as a feed-forward network (it moves information on one path is the layer of input then the layer of hidden after that the layer of output), CNN (a convolution NN that inserts convolution layers to enhance the feed-forward network capabilities), and RNN (a recurrent NN that has an internal memory). Long short term memory (LSTM) is a type of RNN such that LSTM was developed to solve the RNN problem of vanishing gradient which led to the problem of long-term dependency. LSTM is used in many applications, such as speech recognition, anomaly detection, and handwriting recognition. [63] proposed an MLSTM (Multidirectional LSTM) for forecasting the smart grid stability. [64] proposed a system for calculating the similarity of short text using the LSTM encoder. In 2016, [65] proposed the Manhattan LSTM architecture which is a Siamese deep neural network utilizing two LSTMs to calculate the similarity among a pair of phrases.

## 5 Proposed methodology

**Fig 3:**
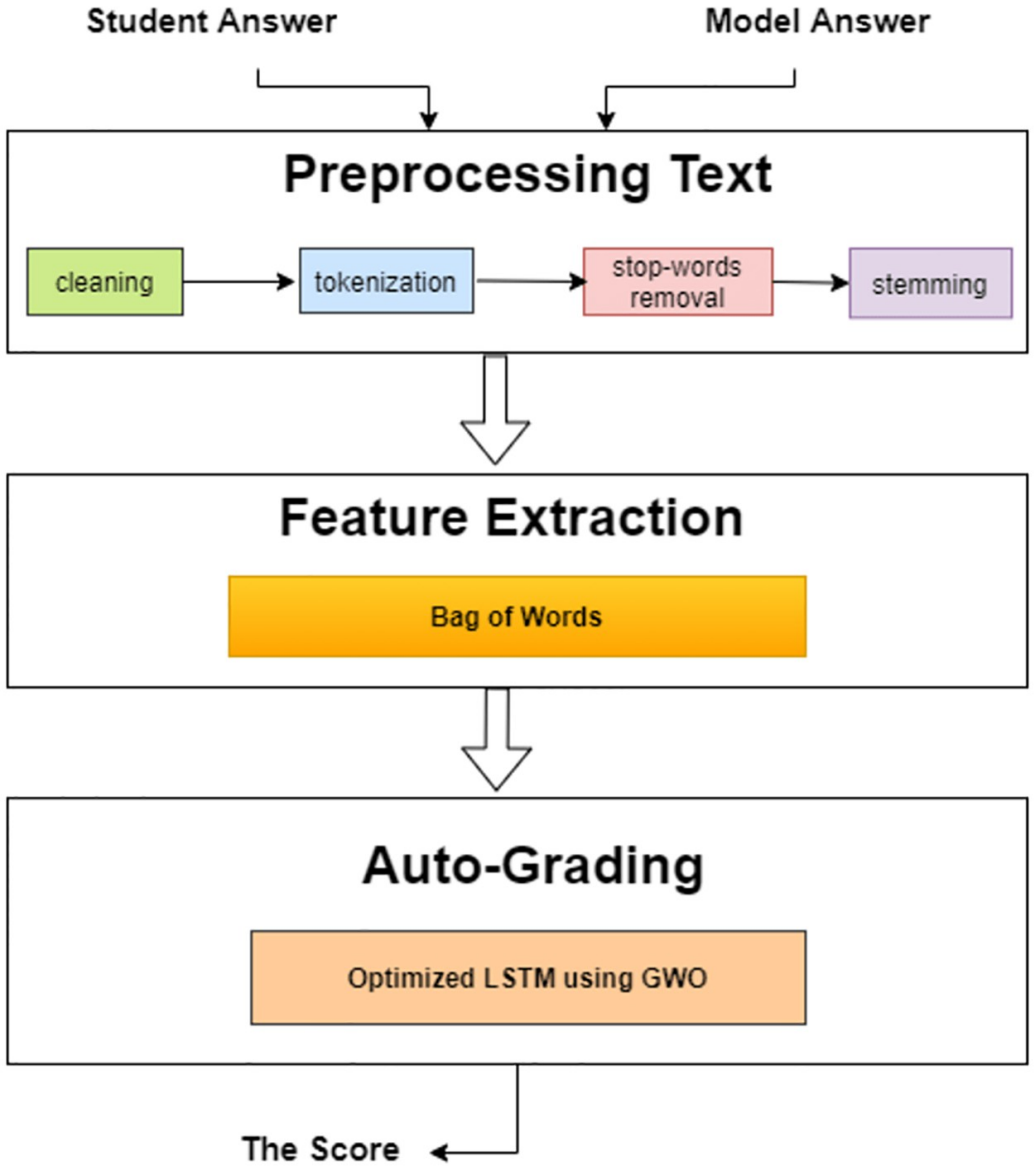
The proposed system block diagram for automatic grading of short responses.

Fig 3 shows the key suggested system architecture which consists of three modules are preprocessing of answers, extraction of features, and feature selection to a hybrid model optimizing LSTM with using GWO to predict the student score, then evaluating the performance accuracy.

### 5.1 Working procedures

The proposed model is executed through three processes are preprocessing data, feature extraction, and Auto-grading.

#### 5.1.1 Preprocessing data

The data is read from two xlsx files; one for the questions and their model answers, and the other contains the students’ answers and the grades given by the human. Three attributes (columns) are selected, which are:

- qno: represents the question number.
- answer: represents the answer text.
- grade: represents the score given by the teacher to the student answer.

For standardization, the first step after the text input is to clean the text by removing and replacing punctuation and some characters such as (إ, ؤ, ئ) in the answers with spaces. For example, the student’s answer:

لأن كثافة الهيليوم والهيدروجين أقل من كثافة الهواء فترتفع لأعلى

The result after cleaning:

ل ن كثافة الهيليوم والهيدروجين قل من كثافة الهوا فترتفع ل على

Preprocessing of data includes: tokenization, stop-words removal, and stemming. The preprocessing steps were processed by the previous result as follows:

1. **Tokenization**
  Through this step, split the text into tokens (words), such as the result will be:
  ’ل’, ’ن’, ’كثافة’, ’الهيليوم’, ’والهيدروجين’, ’قل’, ’من’, ’كثافة’, ’الهوا’, ’فترتفع’, ’ل’, ’على’
2. **Stop-words removal**
  To get high efficiency, we will focus on the important words by removing useless words that are called stop words, such as pronouns and prepositions which have no additional meaning in the student’s answer. Some of the Arabic language stop words are:
  ’إذ’, ’إذا’, ’أكثر’, ’اللذين’, ’إلى’, ’إليك’, ’إليكم’, ’أن’, إنا’, ’أنا’, ’أنت’, ’أنتم’, ’أنتما’, ’أنتن’, ’إنما’,
  By applying the stop words removal technique on the previous tokenization text, the result is:
  ’ل’, ’ن’, ’كثافة’, ’الهيليوم’, ’والهيدروجين’, ’قل’, ’كثافة’, ’الهوا’, ’فترتفع’, ’ل’
3. **Stemming**
  The stemming algorithm aims to convert words to their roots. The ISRI Stemmer was used by the NLTK python library to eliminate possible suffixes and prefixes to give words their base form. The previous result after the stemming process:
  ’ل’, ’ن’, ’كثف’, ’هيليوم ’, ’ميدروج’, ’قل’, ’كثف’, ’هوا’, ’رفع’, ’ل

#### 5.1.2 Feature extraction

Extraction of features converts the pre-processed short answers into numerical forms such as vectors, which are proper for use in machine learning techniques and deep neural networks. BOW represents the text data in vector space model so it is used in NLP to model texts. BOW was utilized to extract and select the important processing features that are relevant to minimize the error rate and reduce the computational cost such that for all of the answers’ unique words, a vocabulary has been created, then each short answer is represented by a numeric vector regards the absent or present of the answer’s words in the vocabulary and regardless the order of words so called bag of words (BOW).

#### 5.1.3 Auto-grading

**Fig 4:**
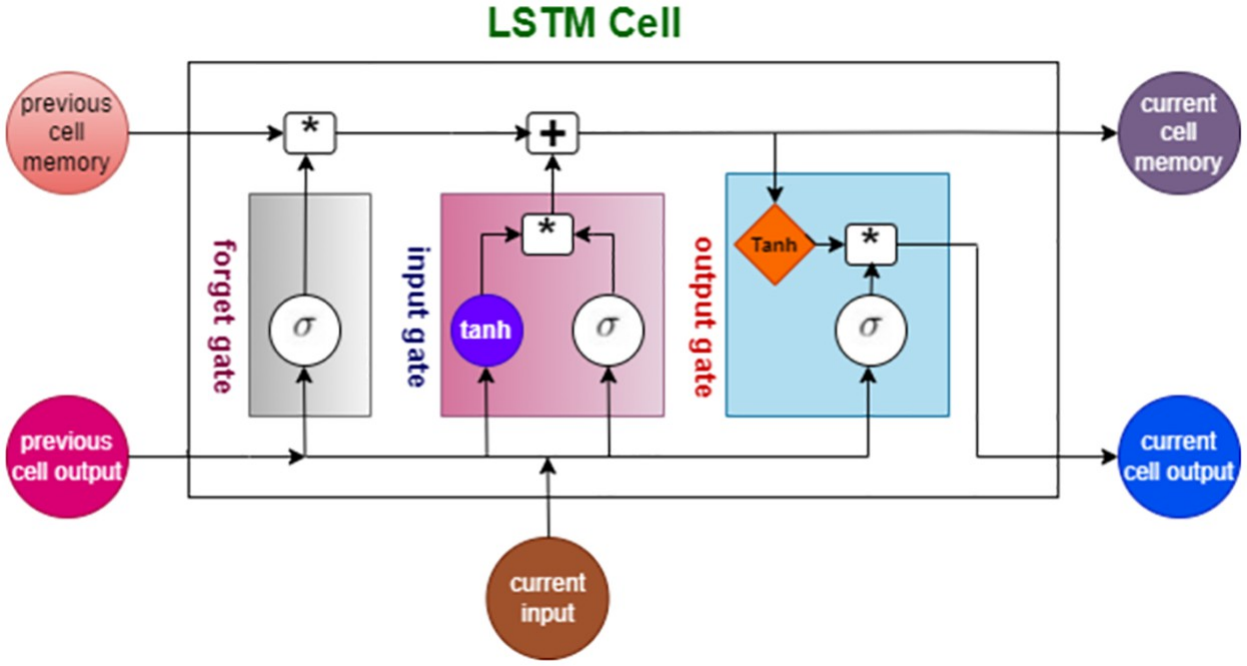
The LSTM cell structure.

The main objective of the research is to predict the student’s score based on the student’s answer to a given question. To obtain more accurate results with less training time, we utilized swarm intelligence algorithms such as GWO to select the best hyperparameters of the LSTM model instead of building a more complex LSTM architecture. The structure of an LSTM cell is illustrated in Fig 4, where the forget gate determines which information is forgotten and which is kept by operating Eq 2 such that the current input and the previous cell output are passed through the sigmoid function, which gives values between 0 and 1, where the output closer to 1 is necessary, while the input gate updates the cell state through operating Eq 3 to pass the current input and the previous cell output to the sigmoid function and operating Eq 4 to pass the same current input and previous cell output to the tanh function, which generates values between -1 and 1. Eq 5 concatenates the point-by-point multiplication of the input gate output of the sigmoid function into the input gate output of the tanh function and the point-by-point multiplication of the forget gate output into the previous cell memory to create a new cell state. Finally, the output gate processes Eq 6 to pass the current input and the previous cell output into the sigmoid function, while the new cell state (current cell memory) is passed through the tanh function. These two functions’ outputs are multiplied point-by-point by processing Eq 7 to generate the current cell output.

$$\begin{array}{c} fo_{t}=\sigma \left(W_{fo}\left[hd_{t-1}\right]+H_{fo}\left[ip_{t}\right]+b_{fo}\right) \end{array}$$ (2)

$$\begin{array}{c} in_{t}=\sigma \left(W_{in}\left[hd_{t-1}\right]+H_{in}\left[ip_{t}\right]+b_{in}\right) \end{array}$$ (3)

$$\begin{array}{c} co_{t}=\text{tanh}\left(W_{co}\left[hd_{t-1}\right]+H_{co}\left[ip_{t}\right]+b_{co}\right) \end{array}$$ (4)

$$\begin{array}{c} cs_{t}=fo_{t}*cs_{t-1}+in_{t}*co_{t} \end{array}$$ (5)

$$\begin{array}{c} ou_{t}=\sigma \left(W_{ou}\left[hd_{t-1}\right]+H_{ou}\left[ip_{t}\right]+b_{ou}\right) \end{array}$$ (6)

$$\begin{array}{c} ch_{t}=ou_{t}*\text{tanh}\left(cs_{t}\right) \end{array}$$ (7)

Where t is the time step, *hd*_*t*−1_ is the previous cell output, *ip*_*t*_ is the current input at t, *W* and *H* are the weight matrices for the forget gate *fo*_*t*_, the input gate output of sigmoid function *in*_*t*_, the input gate output of tanh function *co*_*t*_, and the output gate *ou*_*t*_, while *b* is the bias vector, *cs*_*t*_ is the current cell memory, and *ch*_*t*_ is the current cell output.

To achieve better performance in our proposed system, we will use the Rectified Linear Unit (ReLU) activation function because it returns the input directly if it is greater than 0 and returns 0.0 when the input is negative, so ReLU is a nearly linear activation function and it has a simpler calculation than sigmoid and tanh, which are nonlinear activation functions such that sigmoid generates values between 0 and 1, which transforms the input into 0 if it is less than 0 and to 1 if it is greater than 1, while the tanh activation function returns output between -1 and 1. The ReLU activation function allows easy training of the LSTM network and fast learning. Moreover, ReLU and LSTM can be used to overcome the vanishing gradient problem, which is a special problem in RNN such that unrolling the network for every input time step creates many layers that require updating weights through a back-propagation process to minimize the error rate, but in some cases, the gradient-error is decreased and vanishing, which causes stop in training because the values of the weights have not been changed. By contrast, the larger gradient-error leads to an exploding gradient problem.

The following subsections give more details about the GWO algorithm and how it was used in optimizing the LSTM hyperparameters to enhance the automatic grading model than using the LSTM model alone.

### 5.2 Grey wolf optimization (GWO)

**GWO** has been suggested to be a meta-heuristic algorithm by [66]. GWO has been used to solve real world complex problems and is applied in many fields, such as [67] used GWO was used with SVM to evaluate the quality of the water and classify the pollution degree of the water using microscopic images of fish liver such that the performance is enhanced than using SVM alone in training images [67]. [68] presented GWO for the pointing applications of Laser beam such that GWO was used in the design of the PID (proportional integral derivative) controller which is used in the FSM (fast steering mirror) configuration, where the used function of the objective was The ITAE (integral of time multiplied absolute error) resulted in the design of a PID controller with GWO that achieved the required accuracy and outperformed the current controller. [69] introduced a detailed review of the GWO in terms of types and applications in various areas. The Gray Wolf hierarchy of the leaders and the nature-style hunting mechanism is stimulating the GWO. Gray wolves live in organized groups with pack sizes from 5 to 12. Gray wolf hunting steps are looking for a victim, circle and harass it until it stops moving, and attacking the victim. For the simulation of management hierarchy, four kinds of grey wolves are used, such as:

1. **Alpha wolf**: is the leader of the grey wolves and that can be a female wolf and a male wolf. The responsibilities of the alpha are deciding the sleeping place, time to wake up, hunting, and so on.
2. **Beta wolf**: is the grey wolves’ hierarchy second level and provides alpha feedback and aids alpha in making decisions and extra activities.
3. **Delta wolf**: is the grey wolves’ hierarchy third level and provides food for the pack and works for the pack in the situation of any risk.
4. **Omega wolf**: is the grey wolves’ hierarchy lowest level and can be caretaker, older wolf, scouts, and hunter.

The design of GWO and accomplishing optimization required the hierarchy of grey wolves’ leadership and the method of hunting to be mathematically modeled such that we take the following into account:

- The alpha (α) represents the finest solving.
- The beta (β) represents the second-best solving.
- The delta (δ) represents the third-best solving.
- The omega (ω) represents the rest of the candidate solving.

Through the GWO procedure, the chasing is driven via α wolf, β wolf, and δ wolf such that these wolves are followed by ω wolves. Thus, when designing the algorithm of GWO, the mathematical model of the grey wolves’ hunting (optimization) technique will be as follows:

1. **Encircling Prey**: When the prey is located, the Alpha, Beta, and Delta wolves will lead the Omega wolves to track and eventually surround the prey. The encircling behavior is described by Eqs 8 and 9.
  $$\begin{array}{c} \vec{G}=|\vec{F}.{\vec{Y}}_{p}\left(z\right)-\vec{Y}\left(z\right)| \end{array}$$ (8)
  $$\begin{array}{c} \vec{Y}\left(z+1\right)={\vec{Y}}_{p}\left(z\right)-\vec{E}.\vec{G} \end{array}$$ (9)
  Where *z* denotes the current iteration, $\vec{E}$ and $\vec{F}$ are parameter vectors, $\vec{Y}\left(z\right)$ denotes a grey wolf’s current location, $\vec{Y}\left(z+1\right)$ denotes a wolf’s new location, and *G* denotes a vector that depends on *Y*_*p*_, which represents the prey location. The vectors $\vec{E}$ and $\vec{F}$ are computed by Eqs 10 and 11.
  $$\begin{array}{c} \vec{E}=2\vec{e}.\vec{m_{1}}-\vec{e} \end{array}$$ (10)
  $$\begin{array}{c} \vec{F}=2.\vec{m_{2}} \end{array}$$ (11)
  Where *m*_1_ and *m*_2_ are random vectors in the period [0, 1] while the vector *e* is a controlling coefficient that decreases linearly from 2 to 0.
2. **Hunting**: It is supposed the best solutions in GWO are alpha, beta, and delta because they get better knowledge about the potential location of the prey, which is due to their strength in the pack. Therefore, the other wolves that are the search agents (including the omegas) are obliged to update their positions randomly around the prey as in the proposed Eq 12.
  $$\begin{array}{c} \vec{Y}\left(z+1\right)=\frac{1}{3}\vec{Y_{1}}+\frac{1}{3}\vec{Y_{2}}+\frac{1}{3}\vec{Y_{3}} \end{array}$$ (12)
  where $\vec{Y_{1}}$, $\vec{Y_{2}}$, and $\vec{Y_{3}}$ are computed by Eq 13.
  $$\begin{array}{c} \begin{array}{c} \vec{Y_{1}}=\vec{Y_{\alpha }}-\vec{E_{1}}.\vec{G_{\alpha }},\\ \vec{Y_{2}}=\vec{Y_{\beta }}-\vec{E_{2}}.\vec{G_{\beta }},\\ \vec{Y_{3}}=\vec{Y_{\delta }}-\vec{E_{3}}.\vec{G_{\delta }} \end{array} \end{array}$$ (13)
  The vectors $\vec{G_{\alpha }}$, $\vec{G_{\beta }}$, and $\vec{G_{\delta }}$ have been calculated by Eq 14.
  $$\begin{array}{c} \begin{array}{c} {\vec{G}}_{\alpha }=\left|{\vec{F}}_{1}.{\vec{Y}}_{\alpha }-\vec{Y}\left(z\right)\right|,\\ {\vec{G}}_{\beta }=\left|{\vec{F}}_{2}.{\vec{Y}}_{\beta }-\vec{Y}\left(z\right)\right|,\\ \vec{G_{\delta }}=\left|\vec{F_{3}}.\vec{Y_{\delta }}-\vec{Y}\left(z\right)\right| \end{array} \end{array}$$ (14)
  A search agent can update its location in a search space of n-dimensions based on alpha, beta, and delta through these equations.
3. **Searching and attacking prey**: The search agents have been enabled by the GWO algorithm to upgrade their location and search based on the alpha’s, beta’s, and delta’s location by going in diverse directions to look for the prey and grouping to attack the prey. The **exploration** denotes a wolf that leaves the original search path and seeks a new direction, reflecting the ability of the wolf to exploit unknown areas, whereas the **exploitation** denotes a wolf who continues to investigate more carefully to some degree into the original path, ensuring that the wolf searches in detail in this explored region.
  The proper balance between search (i.e. exploration) and attack (i.e. exploitation) is governed by the random vectors $\vec{E}$ and $\vec{F}$ such that when approaching the prey, the value of the parameter vector $\vec{e}$ should decrease linearly from 2 to 0 and the range of the coefficient vector $\vec{E}$ should change. The next search agent’s position may be in any position between the current position and the location of the prey if a random value of $\vec{E}$ is in [-1,1]. When the value of $\vec{E}$ exceeds 1 and is less than −1, that is $|\vec{E}|>1$, and when the vector value of $\vec{F}$ exceeds 1, the wolf’s search capability may be strengthened. In contrast, if the wolf capacity is increased $|\vec{E}|<1$ and $\vec{F}<1$. To increase the wolf’s ability to exploit (attack), the $\vec{E}$ vector decreases linearly with the increase in iterations. However, the $\vec{F}$ value is randomly generated which can balance wolf exploitation (search) and wolf exploitation in any given phase, so $\vec{F}$ can randomly give the prey weight and make it harder and harder to reach the wolves or vice versa depending on the position of a wolf. It helps GWO in demonstrating a more random behavior, favor exploration, and optimum local avoidance, in particular in the final iterations.

**The GWO algorithm Pseudo Code can be expressed as follow**:

Algorithm 1. The GWO algorithm

- set a maximum number for iterations H
- start the population of the grey wolf *Y*_*i*_(*i* = 1, 2, …, *n*)
- start e, E, and F
- Compute each search agent’s fitness
- *Y*_*α*_ = the search agent of the best
- *Y*_*β*_ = the search agent of the second-best
- *Y*_*δ*_ = the search agent of the third-best
- **while** (*z* < *H*) **do**
   • **for** every search agent **do**
    * Update the current search agent position using previously mentioned equations
   • **end for**
   • Update e, E, and F
   • Compute all search agents’ fitness
   • Update *Y*_*α*_, *Y*_*β*_, and *Y*_*δ*_
   • *z* = *z* + 1
- **end while**
- **return** *Y*_*α*_

### 5.3 Hybrid LSTM-GWO auto-grader

The proposed system is designed with the Grey Wolf Optimizer (GWO) and the Long Short Term Memory (LSTM) network. The GWO algorithm is utilized to optimize LSTM hyperparameters such as the dropout rate and the recurrent dropout rate to predict the student score. The dropout rate and the recurrent dropout rate are used in constructing the LSTM structure, so their values are set before the learning process starts as a regularization method to improve the generalization of the LSTM model. The GWO algorithm selects the dropout and recurrent dropout rates automatically to avoid optimum local and over-fitting problems and improve performance accuracy in the LSTM prediction for the grade of the student in the Arabic short answer questions.

**Fig 5:**
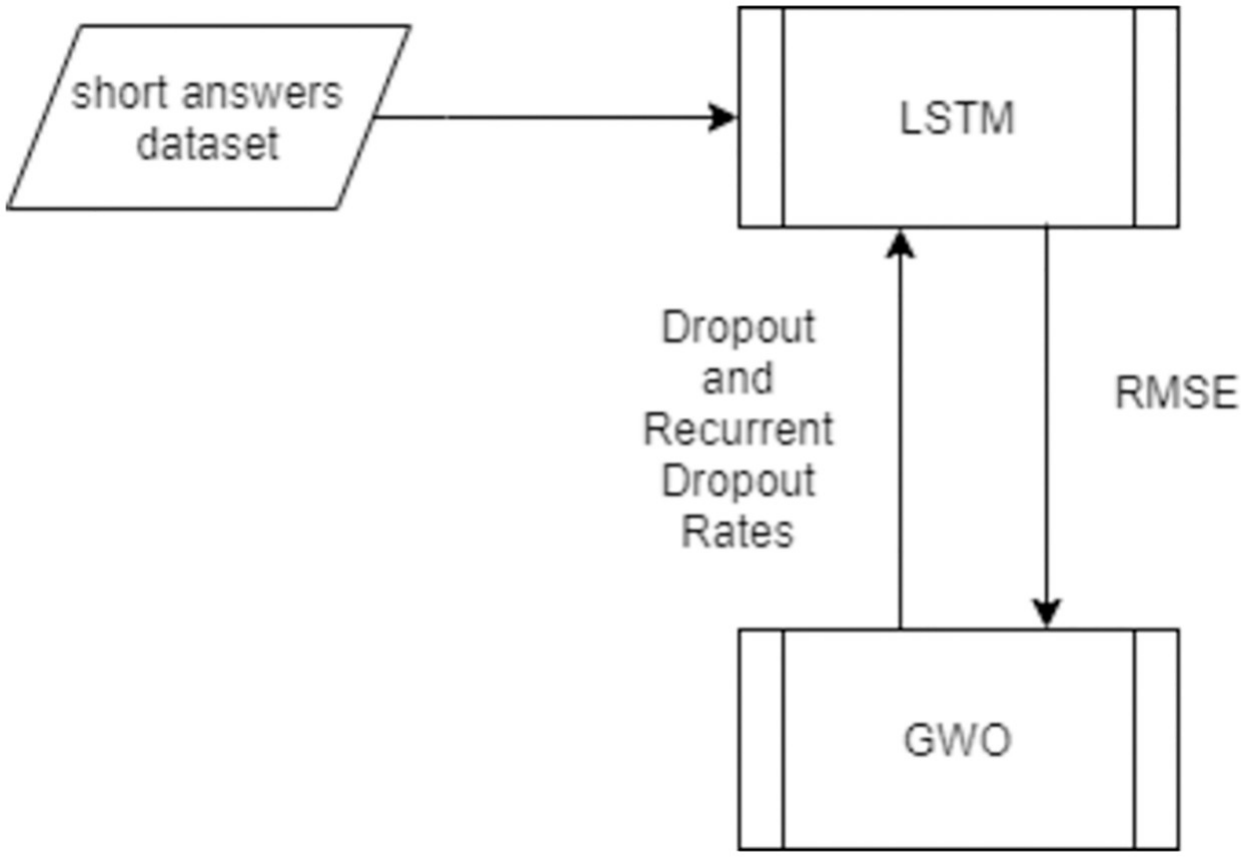
The LSTM optimization process.

Fig 5 shows the optimization process of LSTM using GWO such that the GWO’s objective function is the root mean square error (RMSE), which is computed with Eq 15 through training LSTM on the input dataset. Then the objective function is processed in GWO to get the best values for the dropout and recurrent dropout hyperparameters to be used later in the LSTM model to determine the probability of dropout or exclude the LSTM units of inputs and recurrent connections from updates, thereby preventing the overfitting problem and enhancing the model performance.

$$\begin{array}{c} RMSE=\sqrt{\frac{\sum_{i=1}^{x}{\left(A_{i}-P_{i}\right)}^{2}}{X}} \end{array}$$ (15)

Where for each data point i in X data points, RMSE calculates the square root for the mean of the square of the difference between the prediction output *P*_*i*_ and the actual output *A*_*i*_.

## 6 Experimental results and discussion

### 6.1 Dataset description

In this research, the raw data was gathered from various schools in the Qalyubia-Governorate of the Egypt Arab Republic. The data samples were collected in the Science subject for the 7^*th*^-grade students. The study primarily focuses on the students’ answers for Arabic short answer questions and contained no information that could be used to identify individual students. The students’ answers were graded manually by a teacher such that the given grades range from 0 for fully incorrect response and to 5 for correct response. Table 2 shows a sample of a question, model response, examples of students’ responses, and the grade of the human.

**Table 2:** A sample question, model answer, students’ answers, and grades.

	A Question, Model Answer, and Student Answer	Grade
**Question**	ما هو قانون بقاء الطاقة؟	
**Model Answer**	.الطاقة لاتفض ولا تستحدث من العدم ولكن تتحول من صورة إني ٲخرى	
**Student Answer 1**	.الطاقة لاتفنى ولا تستحدث من العدم	4
**Student Answer 2**	.االطاقة لاتفنى ولاتستحدث بن عدم ولكن تمول من صورة الى صورة ٲخرى	5
**Student Answer 3**	مجموع طاقتى الوضع والحركة لاى جسم	0
**Student Answer 4**	.القوة لاتفنى ولاتستحدث من عدام لكنه	1
**Student Answer 5**	.الطاقة تتحول من صورة اني صورة اخرى	2.5
**Question**	ماذا يحدث عند اكتساب الإلكترون كما من الطاقة؟	
**Model Answer**	.ينتقل الإلكترون إلى مستوى طاقة ٲكلى وتصبح الذرة مثارة	
**Student Answer 1**	.حتى تصبح الذرة مثارة	2
**Student Answer 2**	.ينتقل الالكترون اس مستوى اعلى وتصبح الذرة مثارة	5
**Student Answer 3**	.ينقل	0.5
**Student Answer 4**	.ينتقل لمستوى طاقة أخر	3.5
**Student Answer 5**	.ينتقل اوى مستوى اعلى طاقة	4.5

Table 3 shows Table 2 after translating into English the sample of a question, model response, and examples of students’ responses.

**Table 3:** An English translation of the previous Arabic sample.

	A Question, Model Answer, and Student Answer	Grade
**Question**	What is the law of conservation of energy?	
**Model Answer**	Energy is neither created nor destroyed, but transformed from one form to another.	
**Student Answer 1**	Energy is neither destroyed nor created from nothing.	4
**Student Answer 2**	Energy is neither created nor destroyed, but is transformed from one form to another.	5
**Student Answer 3**	The sum of the potential and movement energies of any body.	0
**Student Answer 4**	Strength is neither destroyed nor created from nothing, but it is.	1
**Student Answer 5**	Energy is transformed from one form to another	2.5
**Question**	What happens when an electron gains energy?	
**Model Answer**	The electron moves to a higher energy level and the atom becomes excited.	
**Student Answer 1**	Until the atom is excited.	2
**Student Answer 2**	The electron moves to a higher level and the atom becomes excited.	5
**Student Answer 3**	move	0.5
**Student Answer 4**	It moves to another energy level.	3.5
**Student Answer 5**	Moves to a higher energy level.	4.5

The dataset is collected in 13 Excel files, additionally to a file contains 50 questions with its model answers. These files are described in Table 4, such that the last and the largest sample contains answers of 300 students on the 50 questions.

**Table 4:** The samples files description.

File	Description	Number of Answers
0	50 Questions with its model answers.	50
1	Answers of 5 questions for 43 students.	215
2	Answers of 50 questions for 27 students, answers of 25 questions for 1 student and answers of the first 5 questions for 15 students.	1450
3	Answers of 50 questions for 17 students, answers of 25 questions for 1 student and answers of the first 5 questions for 25 students.	1000
4	Answers of 50 questions for 11 students and answers of the first 5 questions for 32 students.	710
5	Answers of 50 questions for 40 students and answers of the first 5 questions for 3 students.	2015
6	Answers of 50 questions for 54 students.	2700
7	Answers of 50 questions for 70 students.	3500
8	Answers of 50 questions for 91 students.	4550
9	Answers of 50 questions for 100 students.	5000
10	Answers of 50 questions for 140 students.	7000
11	Answers of 50 questions for 152 students.	7600
12	Answers of 50 questions for 160 students.	8000
13	Answers of 50 questions for 300 students.	15000

### 6.2 Experimental results

**Fig 6:**
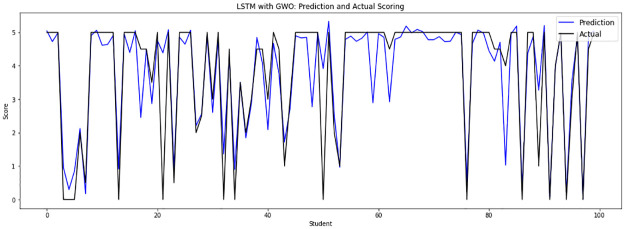
LSTM-GWO prediction and actual scores with experiment 1.

Using various dataset sizes can be considered a contribution emphasizes the accuracy of the results, so we conducted eight experiments with different dataset sizes to perform a comparative analysis and evaluate the effect of using GWO with LSTM in automated short answers. The experiments are described in details as follows:

Experiment 1 drops the duplicated answers from the concatenation of sample files from file 0 to file 12, then adds the sample file 13 to run on 18828 input answers, which are randomly divided into 16945 answers as a training set and 1883 answers as a testing set. Fig 6 shows the experiment 1 prediction score of the LSTM-GWO model and the actual score of the human for the student answer.

**Fig 7:**
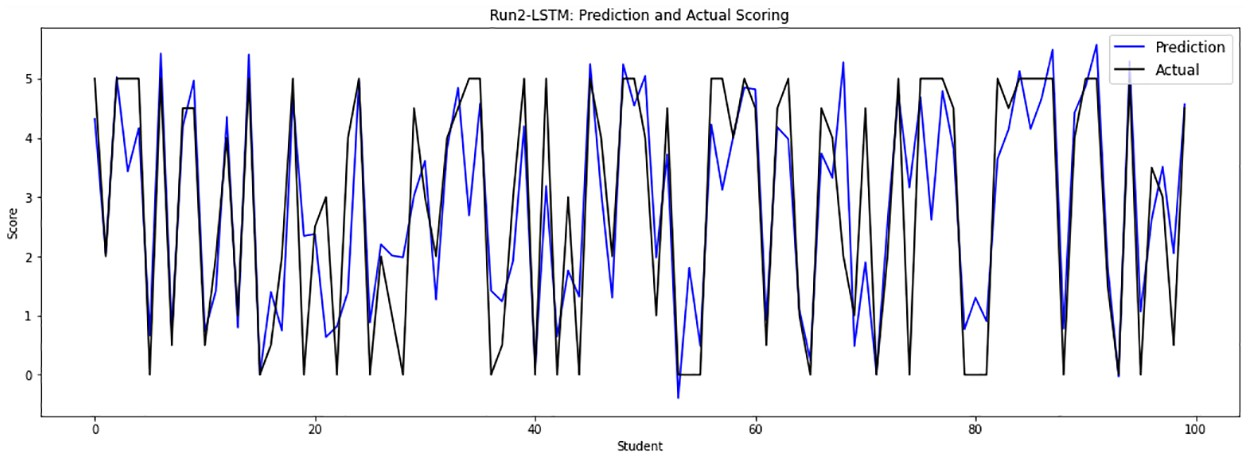
LSTM-GWO prediction and actual scores with experiment 2.

Experiment 2 combines two results: the sample file 13 after dropping the duplicate responses, and the concatenation of sample files from file 0 to file 12 after removing the duplicate answers from files 10, 11, and 12 to run on 8100 answers that are randomly divided into a training set of 7290 answers and a testing set of 810 answers. Fig 7 depicts the real human score and the grade given by the LSTM-GWO model in experiment 2.

**Fig 8:**
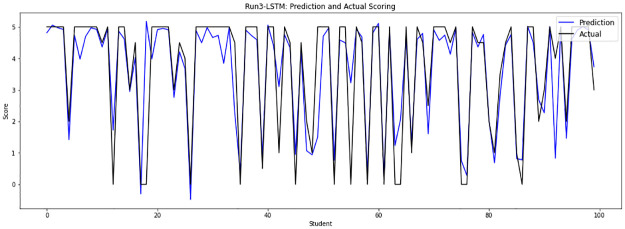
LSTM-GWO prediction and actual scores with experiment 3.

Experiment 3 concatenates the file 0 of model answers with the sample file 13 to implement the 15050 answers, which are randomly divided into a training set of 13545 answers and a testing set of 1505 answers. Fig 8 shows the prediction score plot of the LSTM-GWO model in experiment 3.

**Fig 9:**
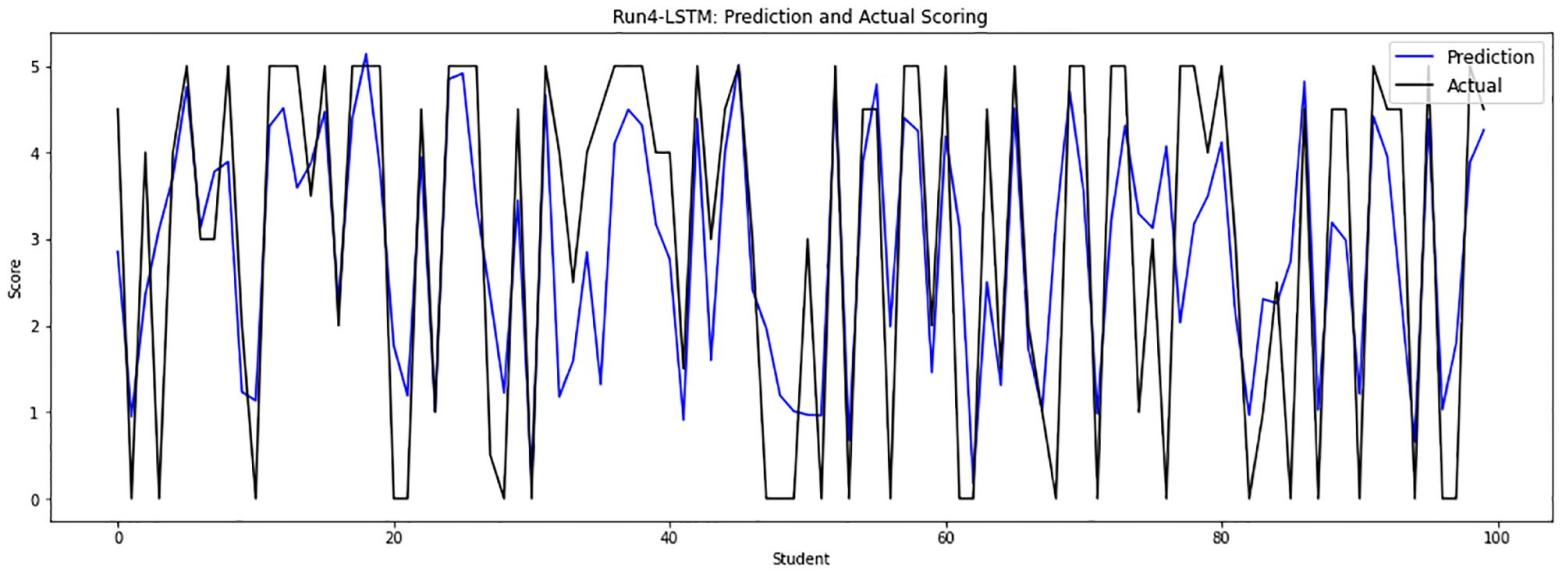
LSTM-GWO prediction and actual scores with experiment 4.

Experiment 4 combines the file 0 of model responses with the sample file 13 after eliminating the repeated responses, implementing answers of 4322 that are randomly divided into a training set of 3889 answers and a testing set of 433 answers. Fig 9 depicts the obtained grades of the LSTM-GWO model through experiment 4.

**Fig 10:**
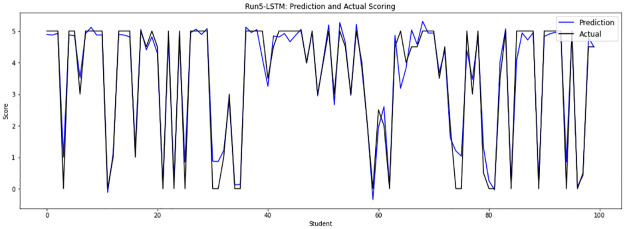
LSTM-GWO prediction and actual scores with experiment 5.

Experiment 5 removes the repeated answers from sample files 10, 11 and 12, then merges them with sample file 13 and files from file 0 to file 9, implementing answers of 47148, which are randomly divided into 42433 answers as a training set and 4715 answers as a testing set. Fig 10 shows the obtained grades of the LSTM-GWO model through experiment 5.

**Fig 11:**
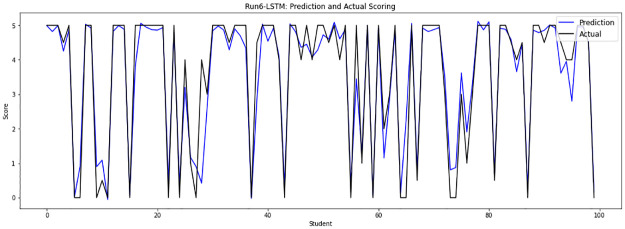
LSTM-GWO prediction and actual scores with experiment 6.

Experiment 6 merges all sample files from file 0 to file 13, running on 58831 answers that are randomly divided into a training set of 52947 answers and a testing set of 5884 answers. Fig 11 shows the prediction score plot of the LSTM-GWO model in experiment 6.

**Fig 12:**
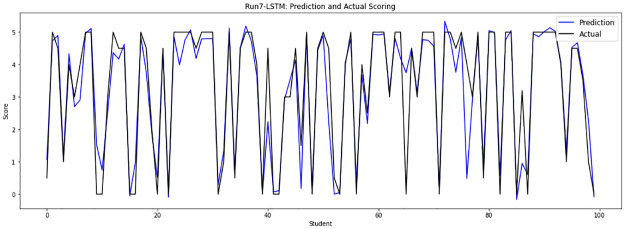
LSTM-GWO prediction and actual scores with experiment 7.

Experiment 7 eliminates the duplicated answers from files 10, 11 and 12, then merges them with sample files from file 0 to file 9, implementing 32148 input answers, which are randomly divided into a training set of 28933 answers and a testing set of 3215 answers. Fig 12 depicts the human grades and the obtained grades of the LSTM-GWO model in experiment 7.

**Fig 13:**
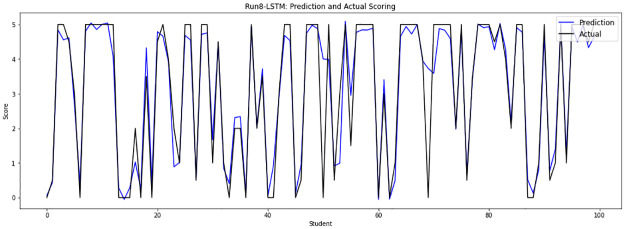
LSTM-GWO prediction and actual scores with experiment 8.

Experiment 8 drops the repeated answers from files 10, 11, 12 and 13, then concatenates them with sample files from file 0 to file 9 to run on 36420 answers that are randomly divided into 32778 answers as a training set and 3642 answers as a testing set. Fig 13 shows the prediction scores by the LSTM-GWO model through experiment 8.

From the graphical representation of the prediction scores, we can see a large convergence between the prediction plot and actual plot in Fig 11 and a convergence between the prediction scores and actual scores in Figs 10 and 12, while some convergence between actual and prediction scores in Figs 6 and 13. In some points of the prediction and actual plots, the divergence appears in Figs 8 and 7, whereas the divergence is increased in some areas in Fig 9. So we infer that the proposed system performed well in prediction in experiment 6, while performance decreased in experiment 4.

The proposed hybrid LSTM-GWO model is applied through eight experiments for the student score prediction based LSTM model and GWO algorithm together. Our system was implemented in the Python 3 programming language.

**Fig 14:**
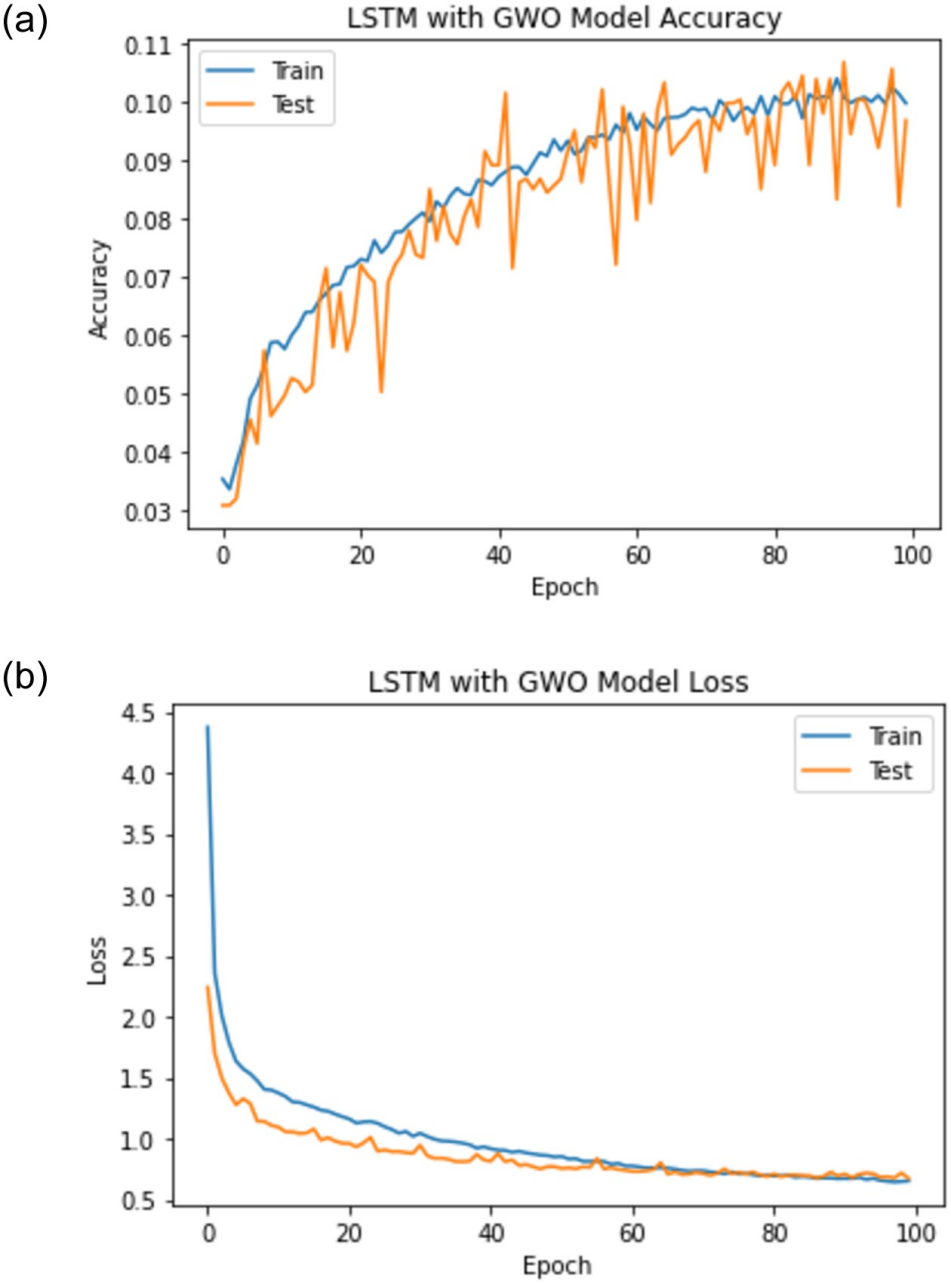
The LSTM-GWO accuracy and loss with experiment 1.

When experiment 1 is run using 18828 answers to predict the student score, it achieves a 0.807 RMSE, a 0.826 R-square, and a 0.909 Pearson r. Fig 14 shows the model accuracy and loss curves with experiment 1.

**Fig 15:**
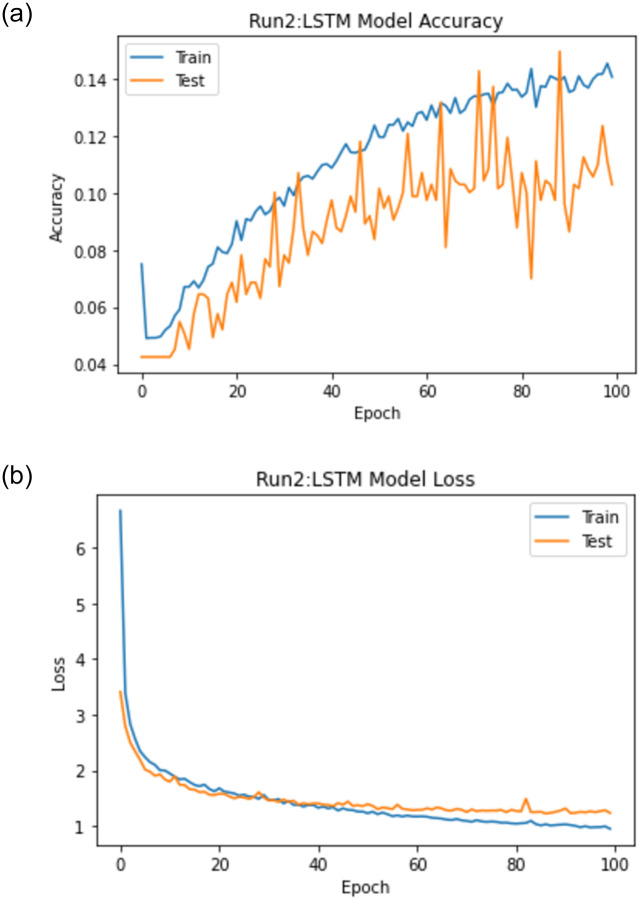
The LSTM-GWO accuracy and loss with experiment 2.

When experiment 2 is run using 8100 answers to predict the student score, it achieves a 1.099 RMSE, a 0.715 R-square, and a 0.846 Pearson r. Fig 15 shows the model accuracy and loss curves with experiment 2.

**Fig 16:**
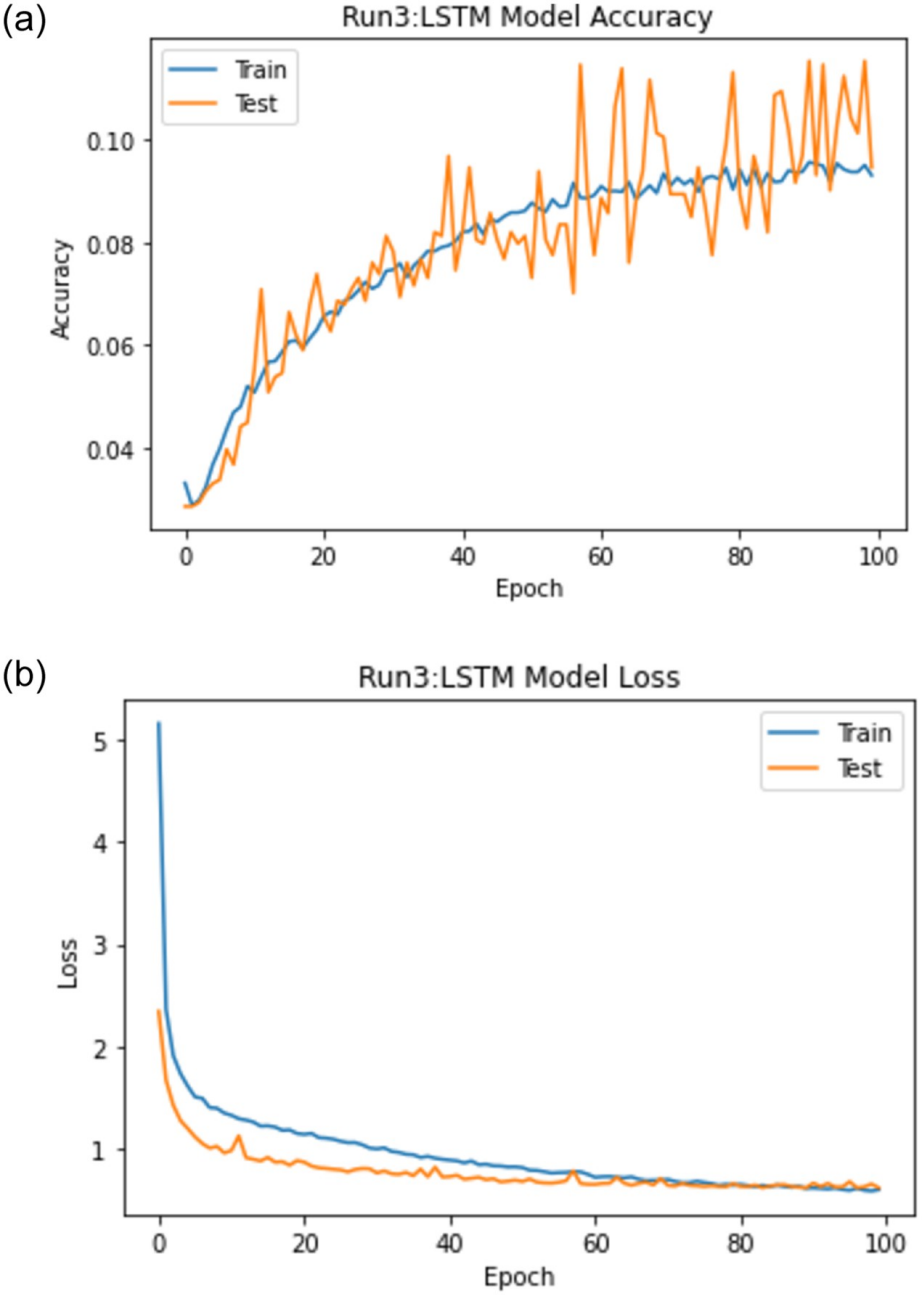
The LSTM-GWO accuracy and loss with experiment 3.

When experiment 3 is run using 15050 answers to predict the student score, it achieves a 0.825 RMSE, a 0.808 R-square, and a 0.900 Pearson r. Fig 16 shows the model accuracy and loss curves with experiment 3.

**Fig 17:**
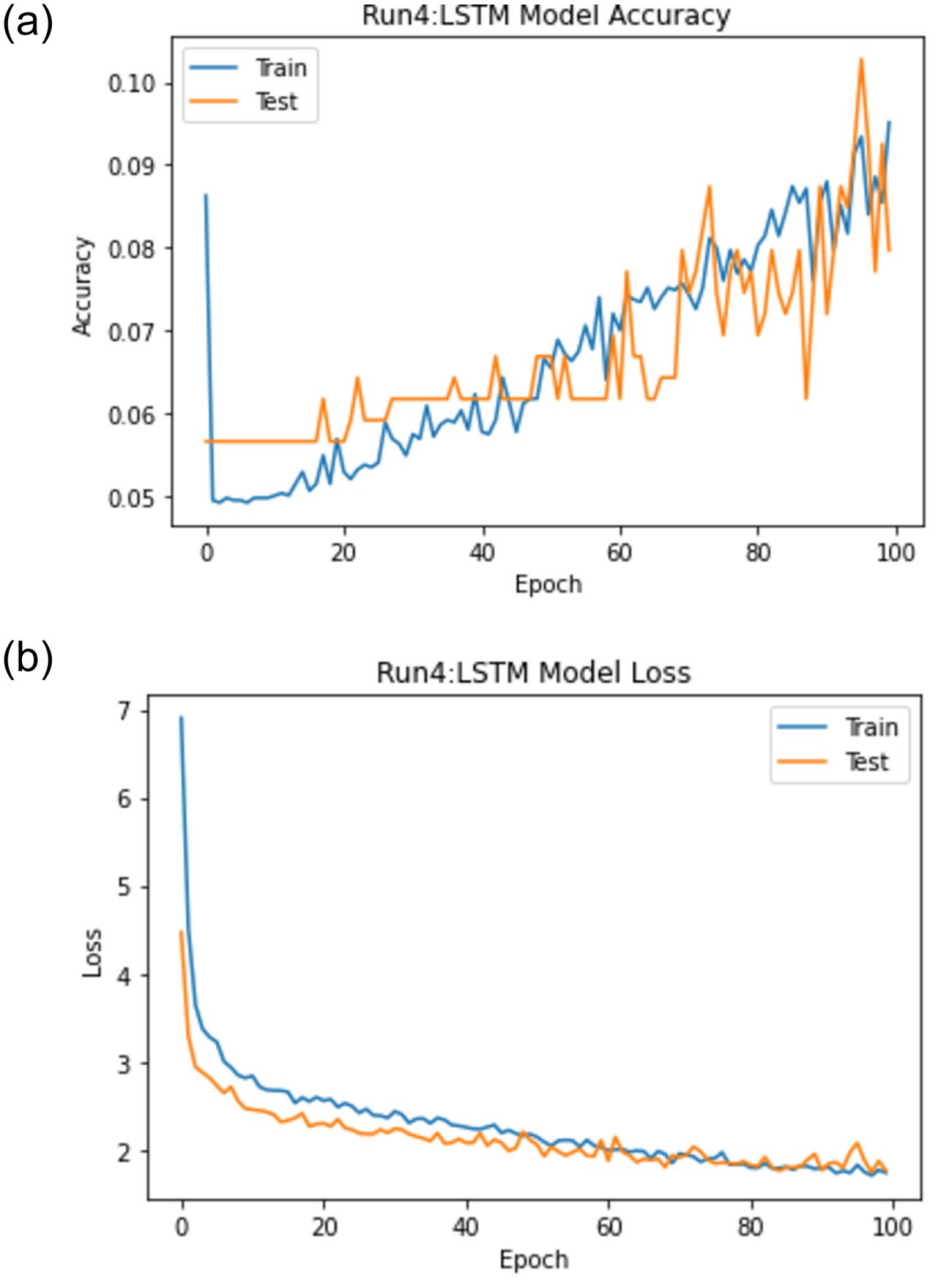
The LSTM-GWO accuracy and loss with experiment 4.

When experiment 4 is run using 4322 answers to predict the student score, it achieves a 1.308 RMSE, a 0.588 R-square, and a 0.772 Pearson r. Fig 17 shows the model accuracy and loss curves with experiment 4.

**Fig 18:**
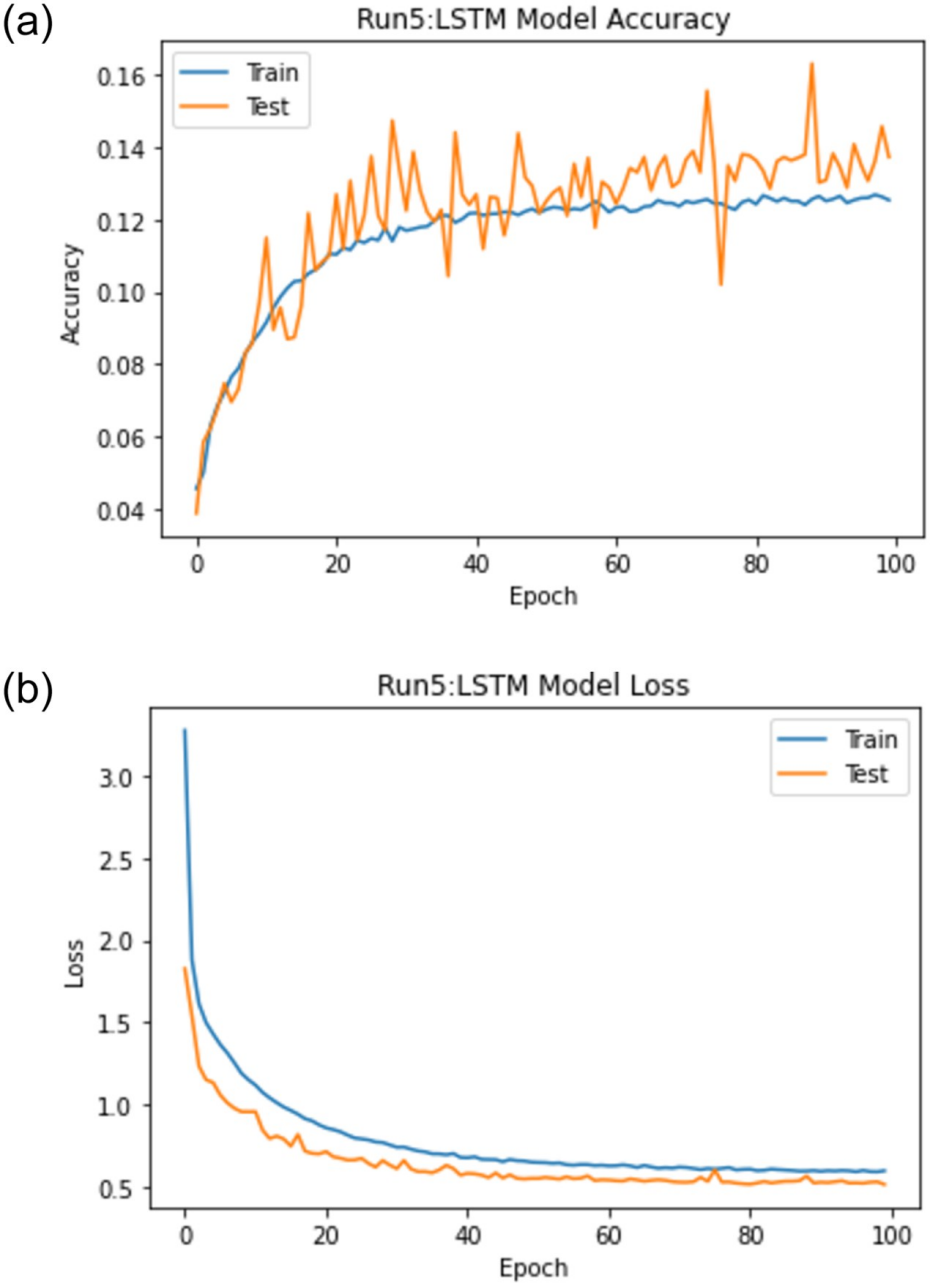
The LSTM-GWO accuracy and loss with experiment 5.

When experiment 5 is run using 47148 answers to predict the student score, it achieves a 0.710 RMSE, a 0.875 R-square, and a 0.936 Pearson r. Fig 18 shows the model accuracy and loss curves with experiment 5.

**Fig 19:**
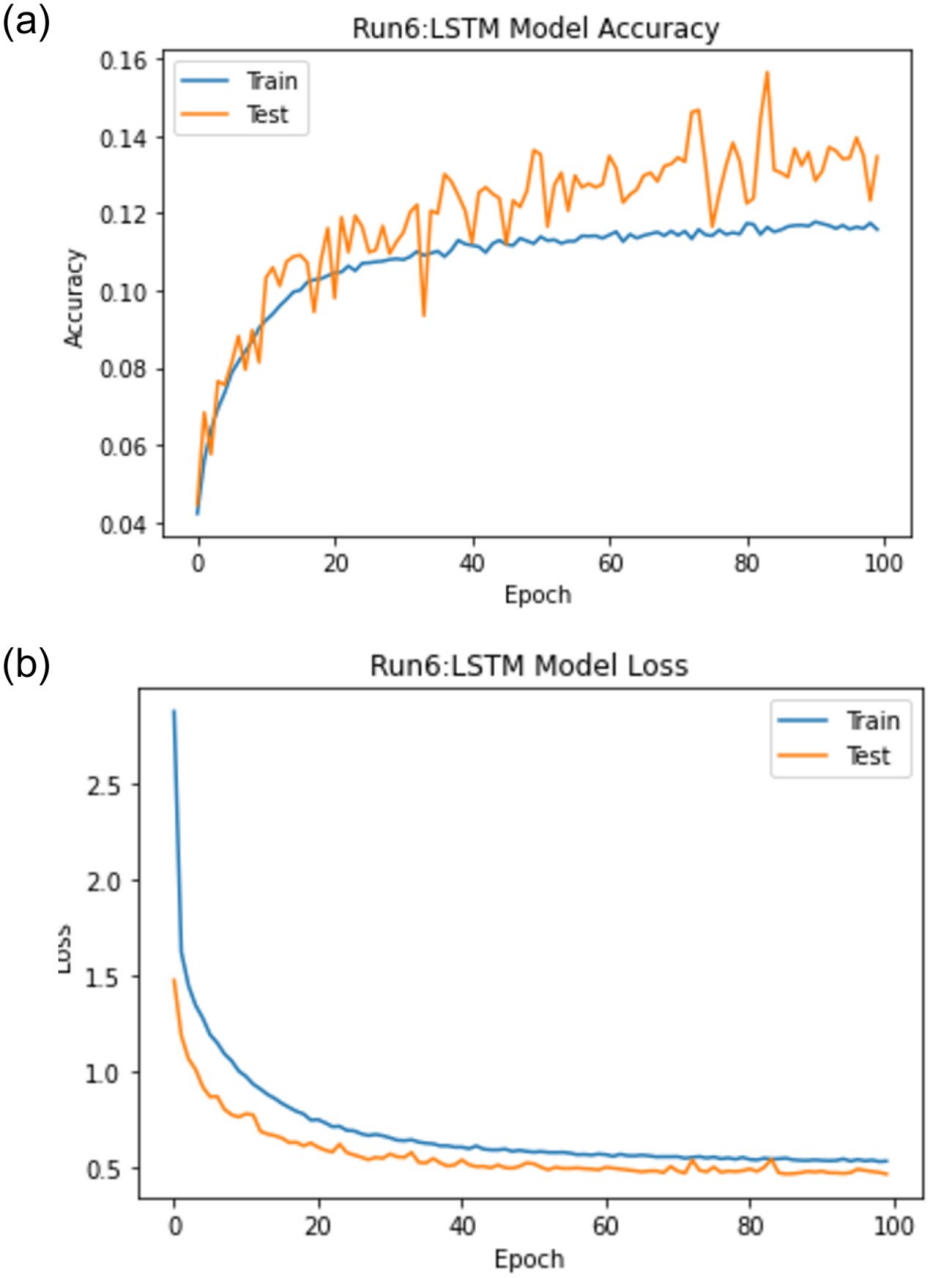
The LSTM-GWO accuracy and loss with experiment 6.

When experiment 6 is run using 58831 answers to predict the student score, it achieves a 0.652 RMSE, a 0.885 R-square, and a 0.941 Pearson r. Fig 19 shows the model accuracy and loss curves with experiment 6.

**Fig 20:**
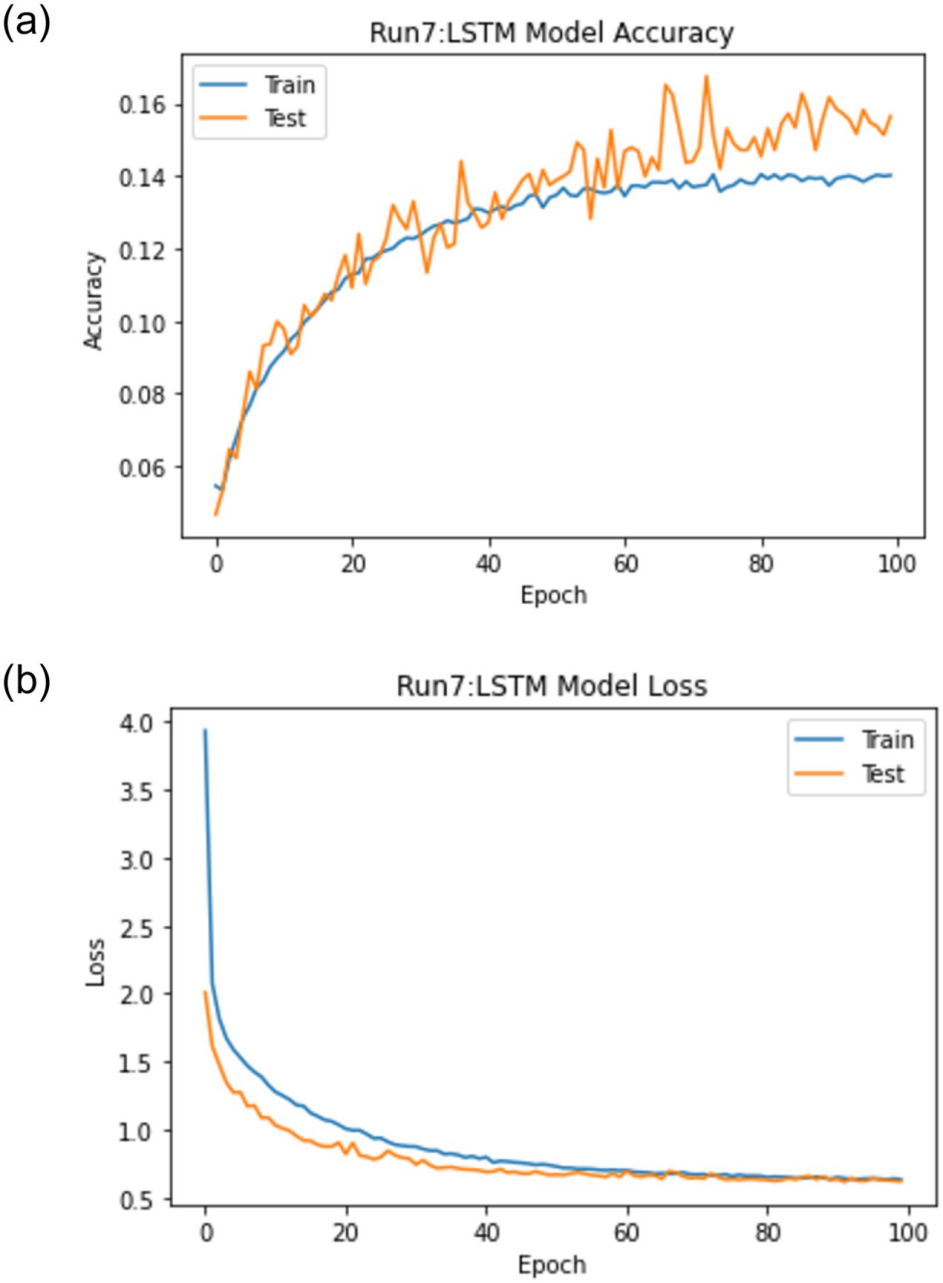
The LSTM-GWO accuracy and loss with experiment 7.

When experiment 7 is run using 32148 answers to predict the student score, it achieves a 0.749 RMSE, a 0.863 R-square, and a 0.930 Pearson r. Fig 20 shows the model accuracy and loss curves with experiment 7.

**Fig 21:**
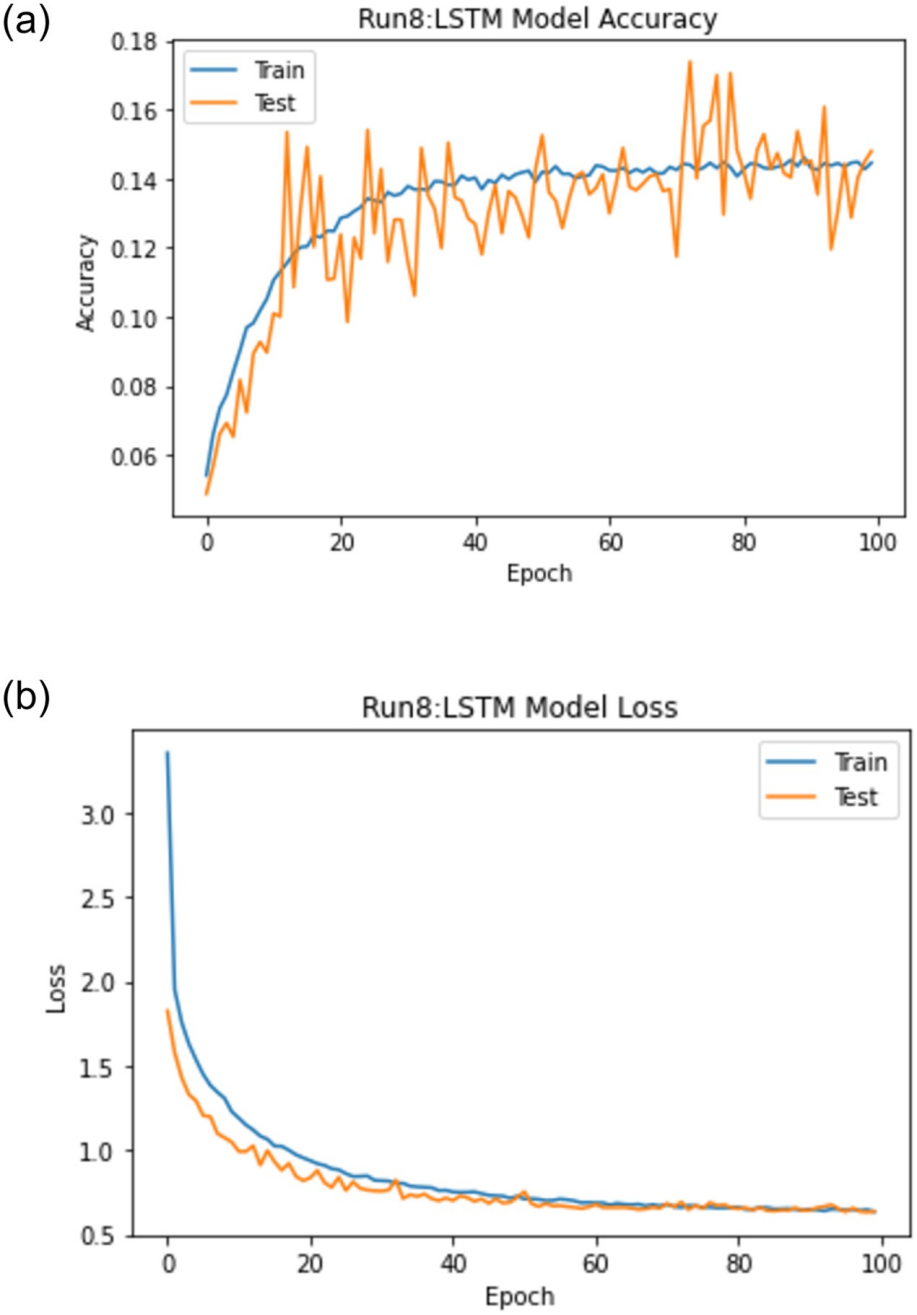
The LSTM-GWO accuracy and loss with experiment 8.

When experiment 8 is run using 36420 answers to predict the student score, it achieves a 0.778 RMSE, a 0.855 R-square, and a 0.925 Pearson r. Fig 21 shows the model accuracy and loss curves with experiment 8.

From the representation of the accuracy and loss of the proposed LSTM-GWO model, we notice that the accuracy increased and the loss error decreased in Figs 18–21, while the accuracy decreased and the loss error increased in Figs 14 and 16, but continuously the low in the accuracy and the high in the loss error are increased in Figs 15 and 17. So we find that the proposed model achieved the highest accuracy and the lowest loss error in experiment 6, whereas there was less accuracy and the most loss error in experiment 4.

Using the same datasets, various experiments are implemented using semantic similarity measurements, LSTM, SVM, and the hybrid SVM with GWO such that:

Importing modules from the Python Keras library is required for building the LSTM model, which includes a sequential model for initializing the neural network and three layers of Dense, LSTM, and Dropout. As hidden layers, two layers of dense are utilized; LSTM model compiled using the popular Adam optimizer. The model was trained in 100 epochs with a 128 batch size.

Building the SVM model requires importing the SVR module of Scikit-learn SVM library in python and creating a support vector regression such that RBF (Radial Basis Function) is used in the kernel to transform a given input dataset into the required form to lead to more accurate results. The SVM model learning outputs by using the fit function on the training set and forecasting the outcomes which are the students’ grades by using the predict function on the testing set.

We used GWO to optimize two variables, C and gamma. These variables are of SVM parameters which affect the learning through the SVM training process where the C parameter or the penalty parameter controls the distance of influence of a single training point, such as the low C values increase the misclassification points and the high C values decrease the misclassification points. However, the gamma parameter determines how far an example of a single training can affect. For instance, the small gamma values denote far while the great gamma values denote close. We applied a GWO algorithm over our dataset to obtain the optimal C and gamma which were used with the RBF kernel to fit the SVM model. The cross-validation of 10-fold is utilised to achieve high accuracy and performance during the validation operation.

This research compares the resemblance between two short texts, which are the answer of the student and the model answer for a particular question based on deep learning and machine learning techniques to calculate the semantic resemblance between the two sentences and assign automatically a grade to the student. Firstly, we calculate the vector of every short text. We built different models for calculating the similarity among the model answer and the student’s answer using the following algorithms: N-gram, Word2vec, LSA, Arabic WordNet, and MaLSTM. All of these techniques have been previously described.

Through the N-gram model, we measure the similitude between the model answer and the student’s answer utilizing a character-based n-gram algorithm by sliding an (*n* = 3) characters window over one string and counting the volume of matches among every substring in the second string and the window. The overall number of matches is split by the whole number of potential n-grams to calculate the uni-directional n-gram similarity uniSim (ans1 -> ans2) from ans1 to ans2 and uniSim (ans2 -> ans1) from ans2 to ans1. The bi-directional similarity is then calculated by averaging the two uni-directional similarities.

In the Word2vec model, word2vec is imported from the Gensim Python module to obtain each word vector in the short response phrase such that as mentioned before we used the Bag of Words technique to create a vocabulary for distinctive words in answers. To evaluate the resemblance of semantic between phrases we use word vectors to obtain sentence vectors by concatenating Min vector, Max vector, and Average vector for each phrase, then use cosine similarity for calculating the similarity between the model answer and the student’s short answer as in Eq 1 where the used vector size is 300 dimensions.

In the LSA model, after cleaning and normalizing, stemming and stop-words removal, we fit two models TF-IDF and SVD in our dataset. The TF-IDF model constructs a Tf-Idf matrix based on unique word frequencies in the dataset. After that, the SVD model takes a matrix returned by the TF-IDF model and performs dimensionality reduction to obtain a concept-by-document matrix. A concept can be considered as a related word series. Then use Eq 1 to calculate the similarity of cosine between the two documents.

**Fig 22:**
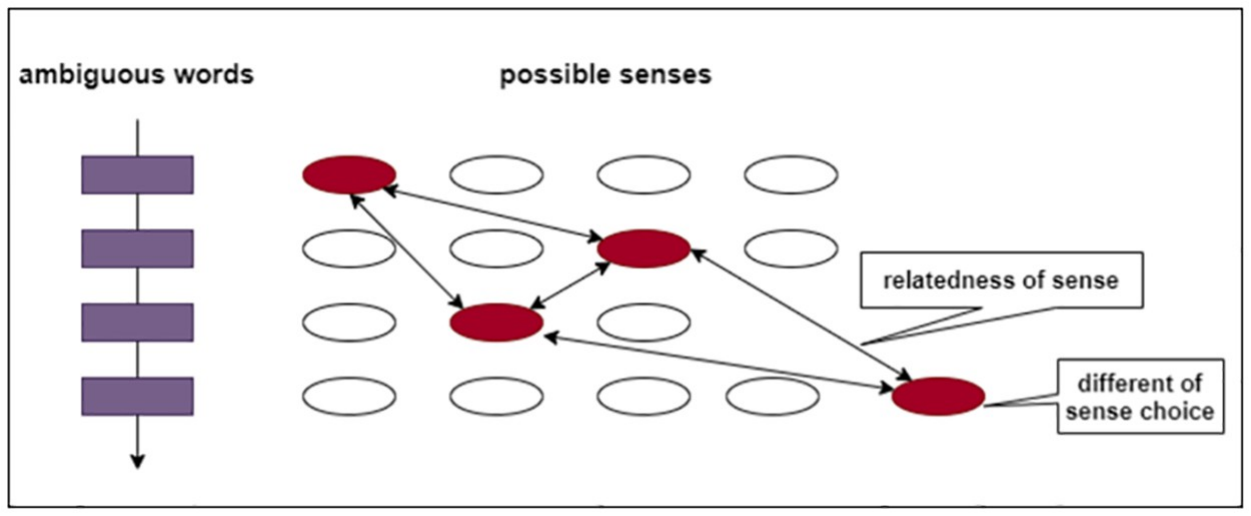
Graphical representation of Lesk.

In the Arabic WordNet model, we used the Lesk measure to remove word ambiguity. Lesk is based on the [70] sensual disambiguation algorithm. POS tagging is employed in the preprocessing text step to identify the words as adjectives, nouns, adverbs, verbs, etc. WordNet for the Arabic language used with Lesk to get all meanings/synsets or each word senses that is a noun, adjective, or verb. Lesk computes the relatedness between pairs of words through finding the overlap among their corresponding definitions of them, which are called glosses that are provided by a dictionary. Fig 22 depicts the Lesk graphical representation which determines the best sense of each of the two words such that the best sense is the one that has the maximum number of mutual words among its definition and the circumstances around the term. After that, we calculate the similitude between the two-word senses to measure the similitude between the words themselves. Wup and Path methods are used for calculating the similarity among the two senses.

MaLSTM (Manhattan LSTM) has been mentioned before as a kind of Siamese network which has two or more identical sub-networks in it. We used two LSTMs networks, one to process the student’s response and the other to process the model’s response. The two LSTMs share the same weight. First, we convert each sentence into a sequence of word embeddings such that every word has been transformed to a vector of fixed-size based on Word2Vec pre-trained in the corpus of GoogleNewsVector. We find the max length of the student’s answer and model answer for padding to make an equal length for both sentences. In the MaLSTM model, the final hidden states of the two layers of LSTM are compared using the Manhattan distance rather than another distance such as Euclidean and Cosine distances. The Manhattan distance is the absolute distance between the final hidden states for the two LSTMs. The Adadelta optimizer is used with gradient clipping to avoid the exploding gradient problem.

### 6.3 Results evaluation

The performance of the proposed hybrid LSTM with the GWO model in auto-grading Arabic short answer questions was evaluated using three measures of evaluation which are:

1. The **RMSE** was employed as a precision metric to compare the prediction errors of different models for a certain dataset. In general, the smaller the RMSE, the closer the fit is to the data.
2. The **R-squared** was used to represent the goodness of fit, where a greater R-squared indicates a better fit for the model in general and the negative value of R-squared indicates that the model does not adequately fit the data.
3. The **Pearson r** was used to determine the linkage among the predicted value and the human-grade, such that Pearson r ranges from −1 to + 1 when near + 1, pointing to a robust positive connection, whereas values close to −1 show a robust negative connection, while values close to 0 show no association.

The proposed LSTM-GWO model was compared with the classical LSTM, SVM, similarity measurements (such as N-gram, LSA, Word2vec (W2V), Arabic-WordNet (AWN), and MaLSTM), and also with the hybrid SVM-GWO.

Table 5 shows the comparison results of the proposed model with compared models in auto-grading over 18828 short answers, such that the best results were marked in bold. As shown in Table 5,the hybrid proposed model LSTM-GWO achieved the best results (less RMSE value, more R-squared value, and high Pearson r value), while the LSA model achieved the worst results (high RMSE value, low R-squared value, and less Pearson r value).

**Table 5:** Comparison of the proposed model and compared models with experiment 1.

Function	LSTM-GWO	LSTM	SVM-GWO	SVM	N-gram	W2V	LSA	AWN	MaLSTM
RMSE	0.807	0.833	1.802	1.991	3.780	2.404	4.037	2.930	2.651
R-Square	0.826	0.813	0.132	-0.067	-2.782	-0.530	-3.315	-1.273	-0.860
Pearson r	0.909	0.902	0.679	0.641	-0.028	0.008	-0.002	-0.008	-0.020

**Fig 23:**
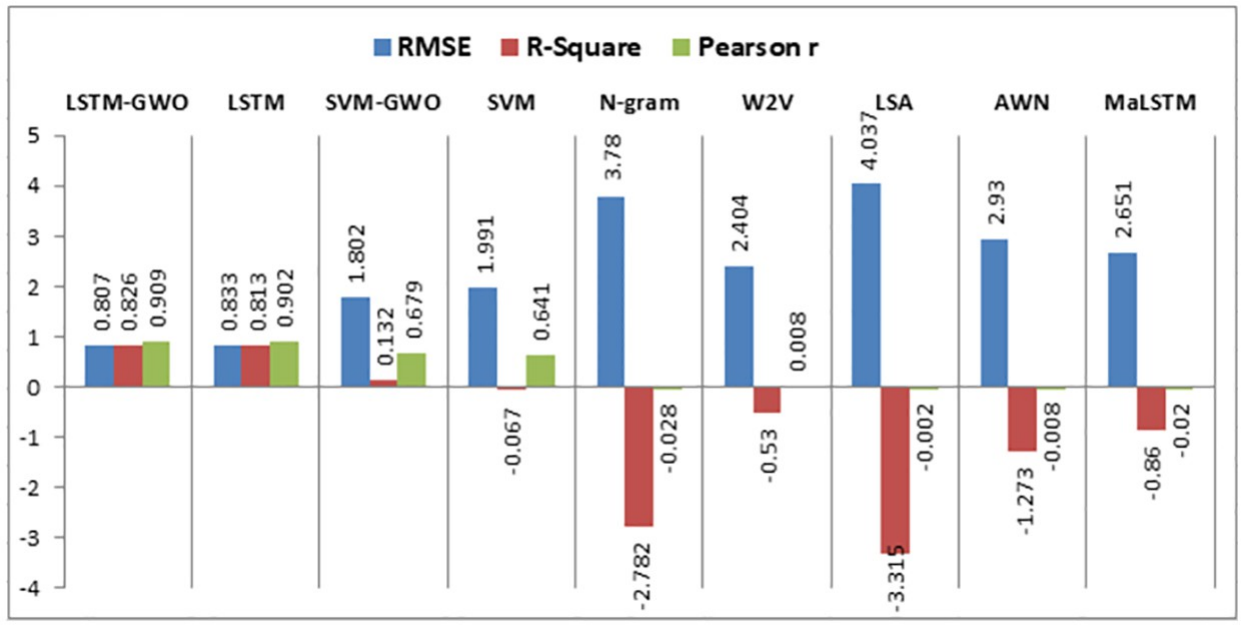
LSTM-GWO performance evaluation with experiment 1.

Fig 23 represents a column chart for the hybrid proposed LSTM-GWO model performance evaluation with other techniques when using the dataset of 18828 answers. Fig 23 transfers the information in Table 5 clearly, such that the blue column represents RMSE, the red column represents R-squared, and the green column represents Pearson r.

Table 6 offers the comparison results of the proposed model with compared models in auto-grading over 8100 short answers.

**Table 6:** Comparison of the proposed model and compared models with experiment 2.

Function	LSTM-GWO	LSTM	SVM-GWO	SVM	N-gram	W2V	LSA	AWN	MaLSTM
RMSE	1.099	1.121	1.636	2.073	3.335	2.842	3.553	2.578	2.883
R-Square	0.715	0.706	0.368	-0.004	-1.647	-0.922	-2.005	-0.582	-0.979
Pearson r	0.846	0.842	0.644	0.478	-0.030	0.004	0.003	0.010	-0.019

**Fig 24:**
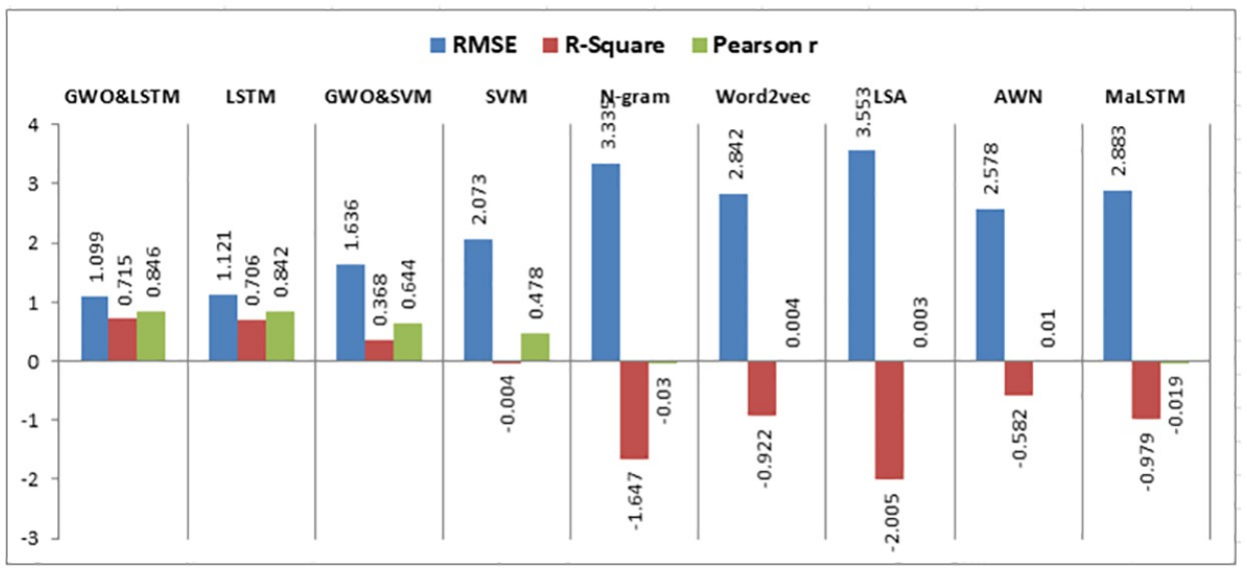
LSTM-GWO performance evaluation with experiment 2.

Fig 24 shows the graphical representation of the proposed LSTM-GWO model performance evaluation with different models when using the dataset of 8100 answers. As shown in Fig 24, the best results were achieved by the proposed LSTM-GWO model, while the worst results were achieved by the LSA model.

Table 7 introduces the comparison results of the proposed model with compared models in auto-grading over 15050 short answers. As shown in Table 7, the proposed model achieved the best results, while the LSA model achieved the worst results.

**Table 7:** Comparison of the proposed model and compared models with experiment 3.

Function	LSTM-GWO	LSTM	SVM-GWO	SVM	N-gram	W2V	LSA	AWN	MaLSTM
RMSE	**0.825**	0.851	1.947	1.994	3.846	2.287	4.113	2.990	2.564
R-Square	**0.808**	0.796	-0.070	-0.122	-3.139	-0.464	-3.733	-1.501	-0.839
Pearson r	**0.900**	0.895	0.664	0.643	0.003	-0.0003	0.007	0.0004	-0.029

**Fig 25:**
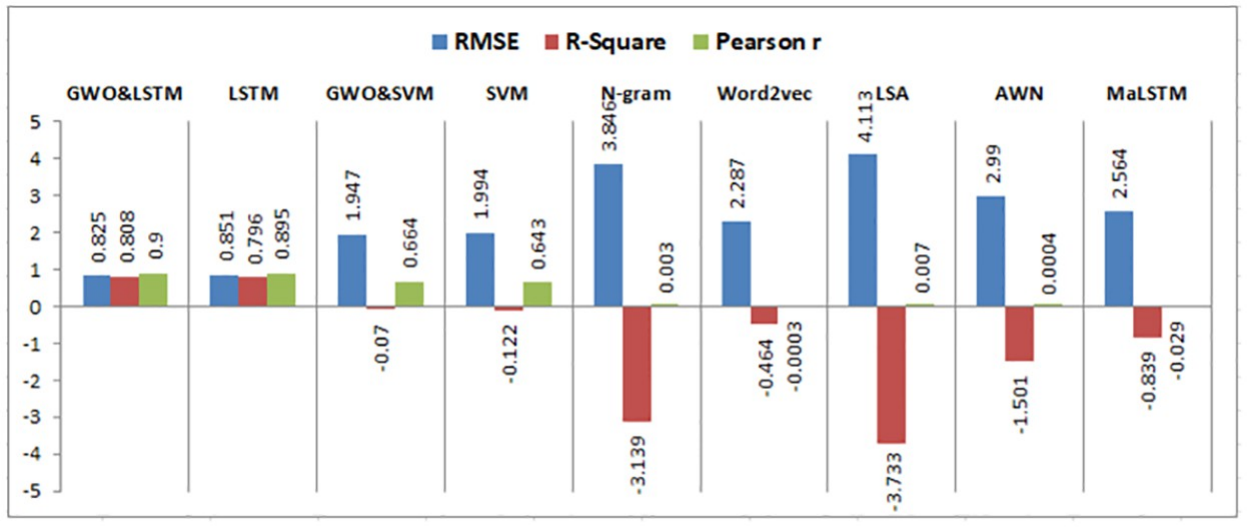
LSTM-GWO performance evaluation with experiment 3.

When using the dataset of 15050 answers, Fig 25 shows the suggested LSTM-GWO model performance compared to other models.

Table 8 shows the auto-grading outcomes of the proposed model versus the compared models over 4322 short answers.

**Table 8:** Comparison of the proposed model and compared models with experiment 4.

Function	LSTM-GWO	LSTM	SVM-GWO	SVM	N-gram	W2V	LSA	AWN	MaLSTM
RMSE	**1.308**	1.422	1.852	2.081	3.320	2.864	3.543	2.574	2.876
R-Square	**0.588**	0.513	0.174	-0.043	-1.598	-0.933	-1.958	-0.562	-0.949
Pearson r	**0.772**	0.740	0.571	0.395	-0.015	0.004	-0.014	0.004	-0.004

**Fig 26:**
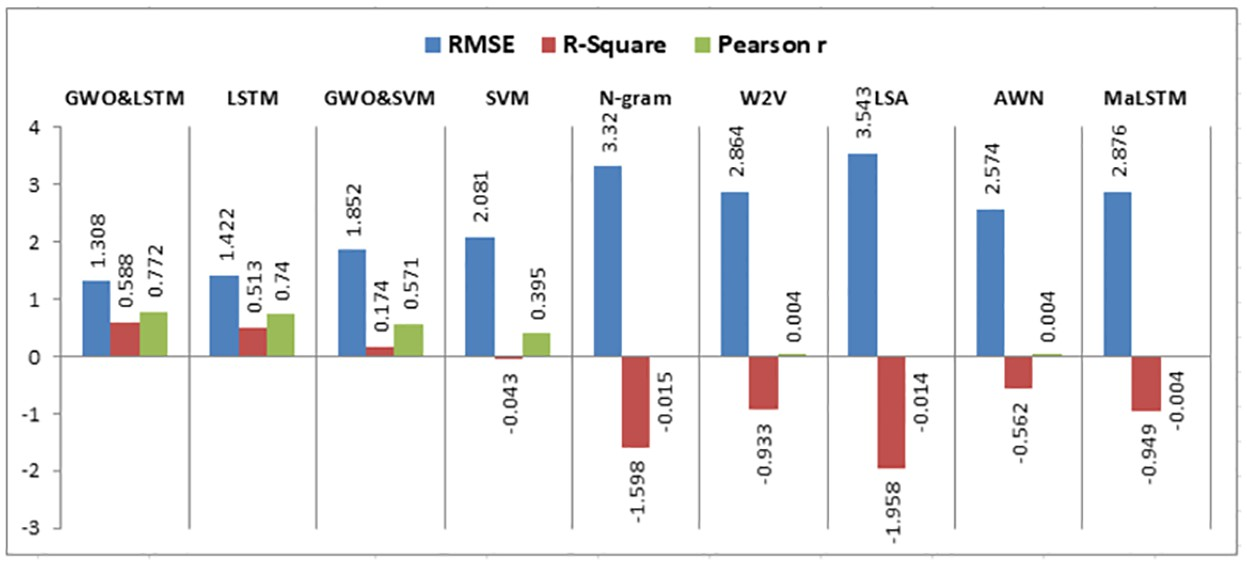
LSTM-GWO performance evaluation with experiment 4.

Fig 26 depicts the proposed LSTM-GWO model performance evaluation with different techniques when using the dataset of 4322 answers. As shown in Fig 26,the proposed model achieved the best results, while the LSA model achieved the worst results.

Table 9 depicts the comparison results of the proposed model with compared models in auto-grading over 47148 short answers. As illustrated in Table 9, the proposed model achieved the best results, while the LSA model achieved the worst results.

**Table 9:** Comparison of the proposed model and compared models with experiment 5.

Function	LSTM-GWO	LSTM	SVM-GWO	SVM	N-gram	W2V	LSA	AWN	MaLSTM
RMSE	**0.710**	0.722	1.454	1.805	3.613	2.565	3.861	2.808	2.008
R-Square	**0.875**	0.871	0.478	0.195	-2.277	-0.652	-2.741	-0.979	-0.012
Pearson r	**0.936**	0.935	0.751	0.716	0.003	0.001	0.0002	-0.010	0.480

**Fig 27:**
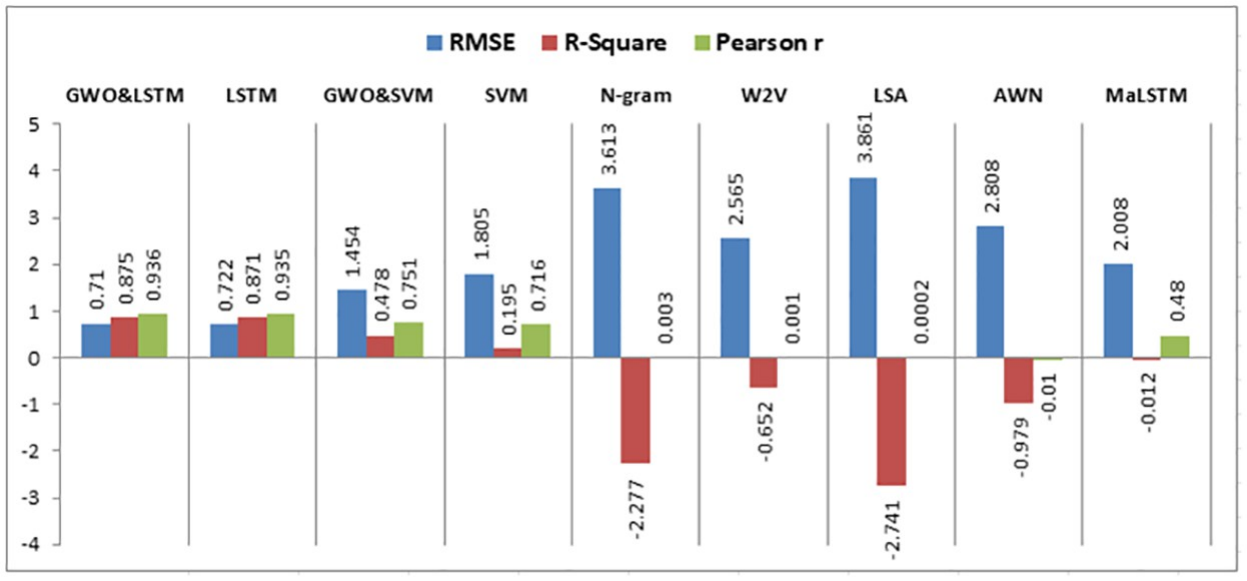
LSTM-GWO performance evaluation with experiment 5.

Using the dataset of 47148 answers, Fig 27 displays the suggested LSTM-GWO model performance evaluation with various methods.

Table 10 introduces the comparison results of the proposed model with compared models in auto-grading over 58831 short answers. As illustrated in Table 10, the proposed model achieved the best results. Although the LSTM model has the same Pearson r value, the proposed LSTM-GWO model produced less RMSE and more R-squared, whereas the LSA model achieved the worst results.

**Table 10:** Comparison of the proposed model and compared models with experiment 6.

Function	LSTM-GWO	LSTM	SVM-GWO	SVM	N-gram	W2V	LSA	AWN	MaLSTM
RMSE	**0.652**	0.673	1.244	1.694	3.735	2.451	3.986	2.895	1.942
R-Square	**0.885**	0.878	0.583	0.226	-2.621	-0.560	-3.126	-1.176	0.021
Pearson r	**0.941**	0.938	0.770	0.714	-0.029	0.009	-0.010	-0.017	0.489

**Fig 28:**
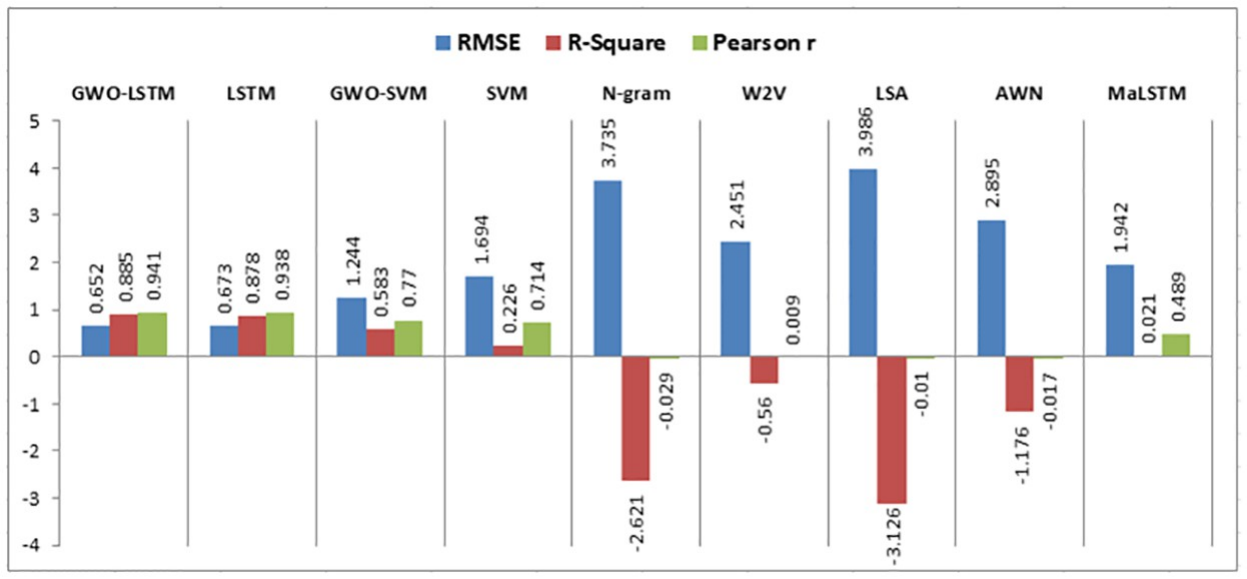
LSTM-GWO performance evaluation with experiment 6.

Fig 28 shows the proposed LSTM-GWO model performance evaluation against other models when using the dataset of 58831 answers.

Table 11 shows the comparison results of the proposed model with compared models in auto-grading over 32148 short answers.

**Table 11:** Comparison of the proposed model and compared models with experiment 7.

Function	LSTM-GWO	LSTM	SVM-GWO	SVM	N-gram	W2V	LSA	AWN	MaLSTM
RMSE	**0.749**	0.768	1.553	1.889	3.490	2.678	3.732	2.701	2.092
R-Square	**0.863**	0.856	0.413	0.131	-1.984	-0.757	-2.412	-0.787	-0.071
Pearson r	**0.930**	0.926	0.721	0.662	0.003	0.004	0.005	0.025	0.527

**Fig 29:**
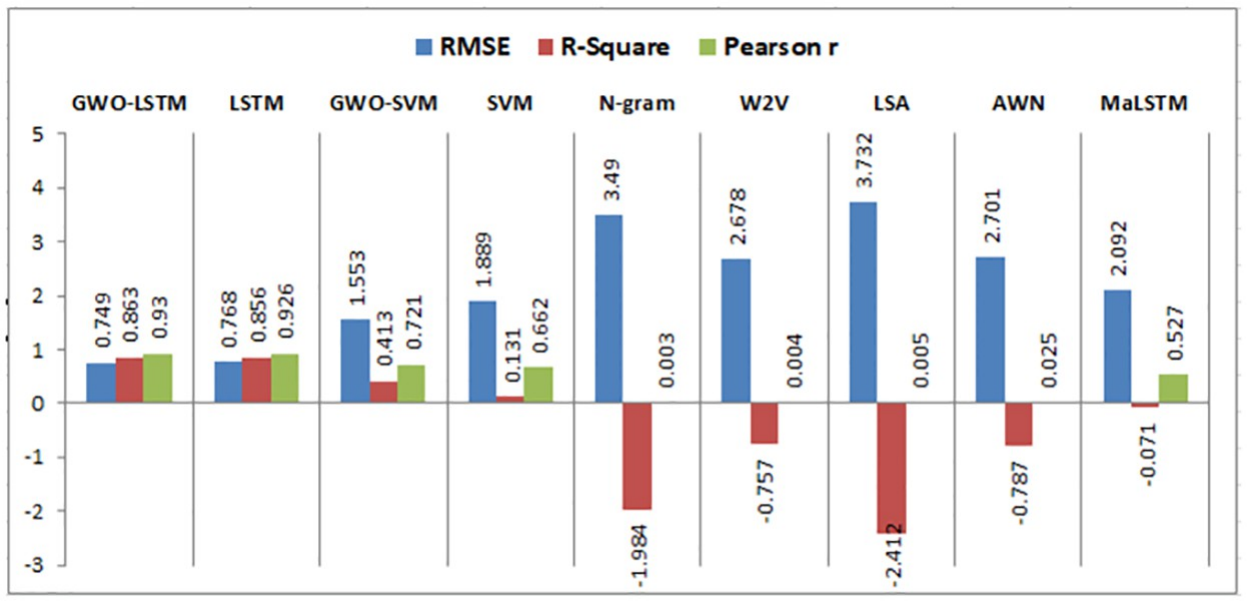
LSTM-GWO performance evaluation with experiment 7.

Fig 29 illustrates the proposed LSTM-GWO model performance evaluation against other techniques when using the dataset of 32148 answers. As shown in Fig 29, the proposed model achieved the best results. Although the LSTM model has the same Pearson r value, the proposed LSTM-GWO model produced less RMSE and more R-squared, whereas the LSA model achieved the worst results.

Table 12 shows the comparison results of the proposed model with different models in auto-grading over 36420 short answers. As illustrated in Table 12, the proposed model achieved the best results, whereas the LSA model achieved the worst results.

**Table 12:** Comparison of the proposed model and compared models with experiment 8.

Function	LSTM-GWO	LSTM	SVM-GWO	SVM	N-gram	W2V	LSA	AWN	MaLSTM
RMSE	**0.778**	0.786	1.495	1.854	3.468	2.699	3.708	2.684	3.223
R-Square	**0.855**	0.852	0.464	0.175	-1.927	-0.773	-2.347	-0.754	-1.529
Pearson r	**0.925**	0.925	0.729	0.665	0.015	0.007	0.114	0.025	0.009

**Fig 30:**
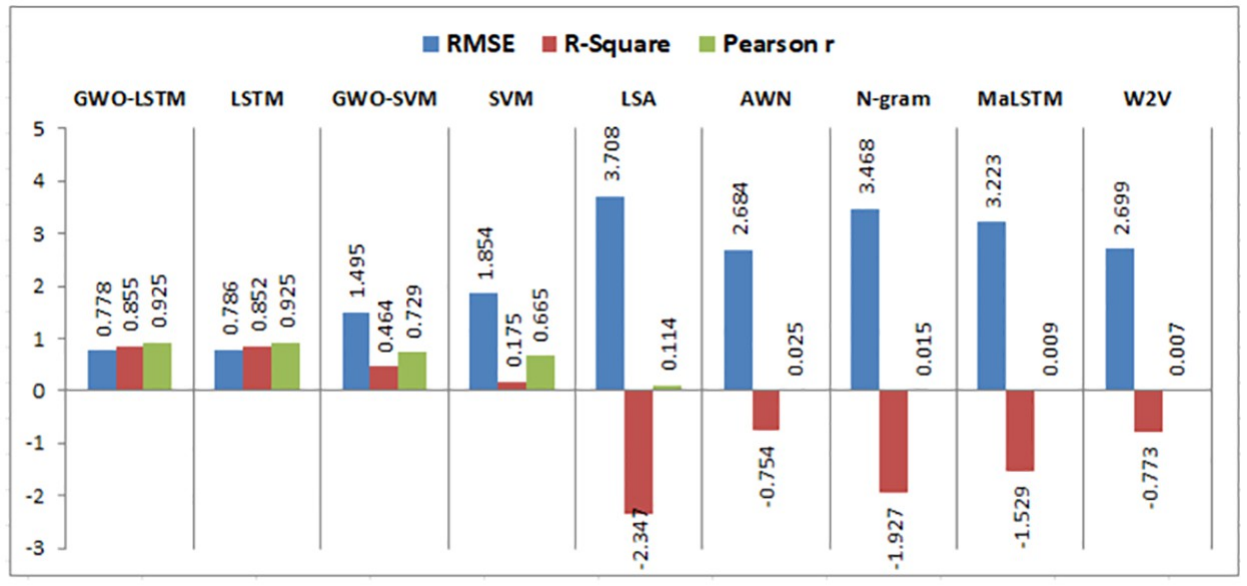
LSTM-GWO performance evaluation with experiment 8.

When using the dataset of 36420 answers, Fig 30 shows the suggested LSTM-GWO model performance evaluation with comparing techniques.

The proposed automatic grading system results when using LSTM with GWO are better than the compared models’ results as shown in tables from Tables 5 to 12 and figures from Figs 23 to 30, where they show that the LSTM-GWO obtained the best results among different models. The proposed model performance was evaluated and compared to the standard LSTM, SVM, N-gram, LSA, Word2vec, Arabic WordNet, MaLSTM, and also hybrid SVM with GWO. In all the experiments, one can notice that the proposed model achieved the lowest RMSE, the best R-square, and the highest Pearson correlation coefficient values. The LSTM was next to the LSTM-GWO and performed better than SVM and SVM-GWO. The LSTM-GWO outperformed the classical LSTM, SVM, and also the SVM-GWO and similarity measurement models.

From the experiments’ results, it is clear that the similarity measurement algorithms achieved poor performance in all experiments, so we drop these models from our comparison and calculate the training time for the rest of the models.

**Fig 31:**
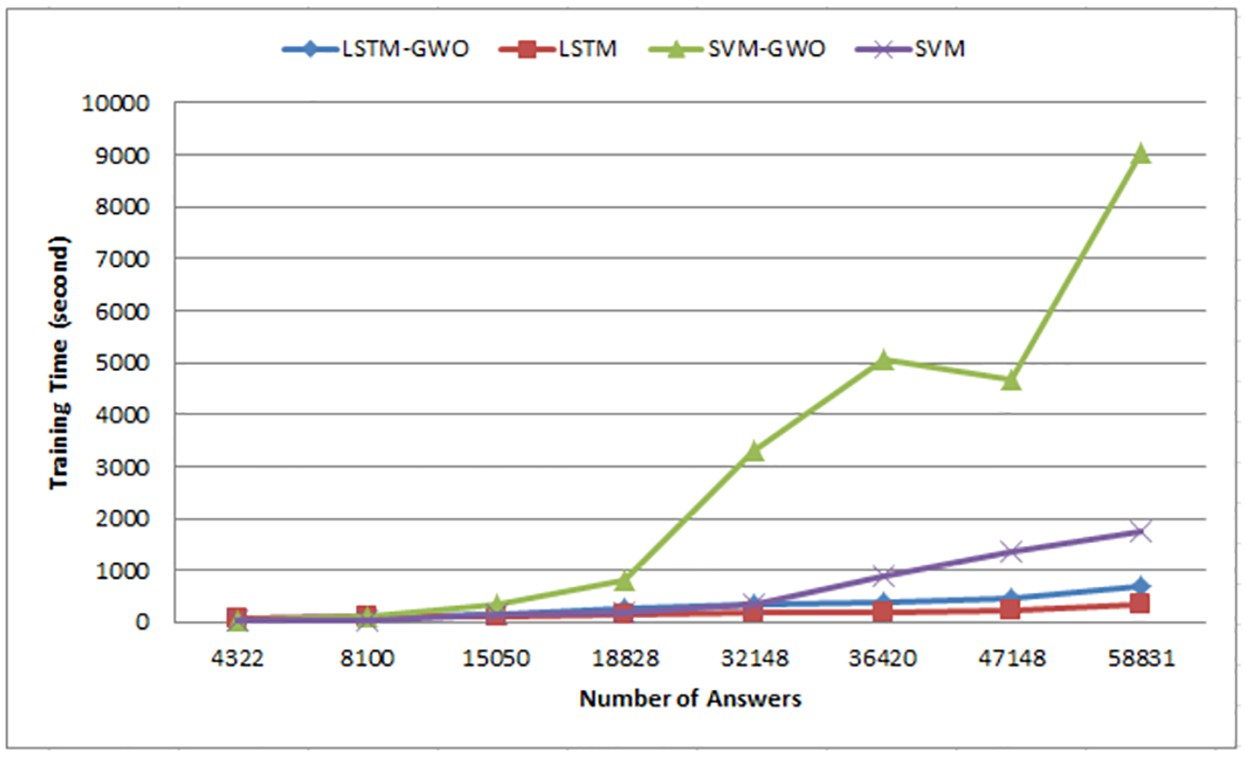
Training time of LSTM-GWO and other models.

Fig 31 shows the training time of LSTM, SVM, SVM-GWO, and LSTM-GWO models.We can notice that the LSTM model had a shorter training time than the other models and was followed by the proposed LSTM-GWO model, which achieved the best results in all experiments with a reasonable training time.

## 7 Conclusion and future work

Automatic grading for Arabic short answer questions has been considered among the most significant learning challenges. In this paper, the hybrid LSTM-GWO model was proposed to predict the student score in Science based on the Arabic short answer of the student for a given question. The prediction technique utilized the GWO in the optimization of the dropout and recurrent dropout rates of the LSTM. Simulation results showed that the hybrid LSTM-GWO model achieved the best accuracy compared to LSTM, SVM, SVM-GWO, N-gram, Word2vec, LSA, Arabic WordNet, and MaLSTM, where the GWO is used to improve the efficiency of the classic LSTM model by choosing the best free parameters. The compared models were evaluated using the RMSE, R-Square, and Pearson correlation coefficient. The study executed eight experiments using various datasets such that in every experiment, the proposed model outperformed compared models by achieving the lowest RMSE value, the best R square value, and the highest Pearson correlation coefficient value, which means the suggested LSTM-GWO model is perfect, more reliable, and produces satisfactory performance in predicting students’ grades, although it has the second order with respect to the training time. Our work had limitations, such as it used classical deep learning and machine learning techniques, so we will use other recent language models, such as BERT and its variants, in our future work. This study was limited to only the auto-grading of the short answers in the Arabic language, so we hope to apply the proposed system to other languages. We will integrate the compared methods to overcome the gaps in each and get better results for future work. Furthermore, more work will be expended on hybrid algorithms based on swarm algorithms.
